# Supplementary material for: CtpB Assembles a Gated Protease Tunnel Regulating Cell-Cell Signaling during Spore Formation in Bacillus subtilis
Source: Cell. 2013 Oct 24;155(3):647–58. doi: 10.1016/j.cell.2013.09.050 (PMC3808539; doi:10.1016/j.cell.2013.09.050)
Supplement: Document S1. Tables S1–S6 [file mmc1.pdf]

Table S1. Data Collection, Phasing and Refinement Statistics, Related to Figure 2

	CtpBa1	*CtpBa2/3*	*CtpBi*
Space group	$P3_221$	$P2_1$	P1
Cell dimensions			
a, *b*, *c* (Å)	118.7, 118.7, 72.6	117.0, 65.3, 169.1	54.1, 72.7, 79.6
α, β, γ (°)	90, 90, 120	90, 95.1, 90	117.1, 90.4, 102.7
**Data collection**			
Wavelength (Å)	0.979	0.979	0.979
Resolution (Å)[a]	40-1.9 (2.0-1.9)	40-2.6 (2.7-2.6)	40-1.8 (1.9-1.8)
R_{sym} (%)	5.8 (67.1)	14.0 (81.2)	4.7 (38.1)
I /sigma(I)	7.5 (1.1)	4.1 (1.4)	11.9 (2.0)
Completeness (%)	100.0 (100.0)	100.0 (100.0)	96.7 (95.4)
Redundancy	10.2 (10.4)	6.8 (6.8)	3.9 (3.9)
**Phasing**			
Phasing power	2.06 (0.35)		
Lack-of-closure	0.58 (0.99)		
Figure-of-merit	0.40 (0.17)		
**Refinement**			
Resolution (Å)	15-1.9	20-2.7	10-1.8
No. of reflections	46645	70460	93030
R_{work}/R_{free}(%)	19.5/21.6	22.7/24.1	18.2/19.1
No. atoms			
Protein	3382	12851	6794
Ligands	41	227	
Water	334	274	985
B-factors (Å^2)			
Protein	41.5	63.2	27.8
Ligand	53.2	56.7	
Water	47.7	46.3	39.4
rms deviations			
Bond lengths (Å)	0.008	0.008	0.006
Bond angles (º)	1.13	1.14	1.00
Ramachandran statistics (%)			
most favored	93.4	92.9	94.3
additional allowed	6.3	6.5	5.4
generously allowed	0.0	0.4	0.1
disallowed regions	0.3	0.2	0.3

[a] Highest resolution shell is shown in parenthesis.

Table S1 (continued). Data Collection, Phasing and Refinement Statistics, Related to Figure 2

	S309A/VPA	*V118Y*	*R168A*
Space group	$P3_221$	P1	$P3_221$
Cell dimensions			
a, *b*, *c* (Å)	118.7, 118.7, 72.4	54.1, 70.9, 77.3	117.9, 117.9, 72.0
α, β, γ (°)	90, 90, 120	63.6, 76.8, 76.5	90, 90, 120
**Data collection**			
Wavelength (Å)	0.979	0.979	0.979
Resolution (Å)[a]	20-1.9 (2.00-1.90)	20-1.95 (2.06-1.95)	20-2.4 (2.53-2.40)
R_{sym} (%)	3.1 (69.6)	10.3 (50.9)	1.4 (33.9)
I /sigma(*I*)	20.3 (0.9)	5.0 (1.3)	45.5 (2.3)
Completeness (%)	98.5 (97.5)	93.9 (84.6)	99.7 (100)
Redundancy	3.2 (3.1)	2.8 (1.8)	2.8 (2.9)
**Phasing**			
Phasing power			
Lack-of-closure			
Figure-of-merit			
**Refinement**			
Resolution (Å)	20-1.9	20-1.95	20-2.4
No. of reflections	45,467	67,456	22,041
R_{work}/R_{free}(%)	20.3/22.5	20.5/23.9	21.5/26.5
No. atoms			
Protein	3381	6806	3376
Ligands	46		53
Water	359	517	70
B-factors (Å^2)			
Protein	47.2	35.0	79.5
Ligand	55.6		100.1
Water	52.3	38.9	63.6
rms deviations			
Bond lengths (Å)	0.007	0.007	0.007
Bond angles (º)	1.31	1.27	1.30
Ramachandran statistics (%)			
most favored	91.7	90.7	84.5
additional allowed	8.1	8.5	15.0
generously allowed	0	0.4	0.3
disallowed regions	0.3	0.4	0.3

[a] Highest resolution shell is shown in parenthesis.

Table S2. Proteins Predicted to Reside in the Interspace of *B. subtilis* Having a Phe/Tyr/Trp-Val/Ala C-Terminus, Related to Figure 4

C-terminus – last residue number	ID	Name	type of protein, indicating last residue of folded portion
**NLVLAGLHSYA-391**	P35162	Cytochrome c biogenesis protein resC	MP[1], last helix until 384
**RLAEYIQQPFV-175**	P80871	General stress protein 14	flavodoxin-like fold, until 171
**YVKKITSVYYA-181**	O31608	Putative murein lytic transglycosylase yjbJ	lysozyme-like fold, until 180
**NILEKKYAHYV-226**	P42399	ABC transporter permease protein yckA	MP, last helix until 216
**TRLEDIISRYV-199**	P81100	Stress response protein SCP2	TerD domain, until 197
**EQANTLFTSYV-189**	P32393	ComE operon protein 2	deaminase domain, until 184
**RQLDEIMNSWA-197**	O34932	Dephospho-CoA kinase	triphosphate hydrolase, until 196
**KKKQLKKTVYL-403**	O07639	Uncharacterized membrane protein ylaO	MP, C-term in cytosol
**LFMLLRRKAYA-316**	P94418	ABC transporter permease protein yclN	MP, last helix until 310
**DIFISLYKDFA-393**	O07587	Putative aspartate aminotransferase yhdR	pyridoxal phosphate enzyme, until 393
**LFIGDVDVKYV-241**	O32199	Protein liaF	DUF2154 domain, until 126-238
**YVGLKAIFAFV-202**	P08064	Succinate dehydrogenase cytochrome b558	MP, last helix until 196

[1]MP: internal membrane protein

Table S3. Sporulation Efficiencies of Indicated *B. subtilis* Strains, Related to Figure 6

strain	CFU[1]/ml ($x10^6$)	spores/ml ($x10^6$)	sporulation efficiency (spores/CFU)	average sporulation efficiency (with SEM)
**WT**	276	226	0.81	**0.80 ± 0.07**
	314	197	0.63	
	341	333	0.98	
	266	212	0.80	
**ΔCtpB**	190	175	0.92	**0.78 ± 0.08**
	219	111	0.50	
	345	307	0.89	
	179	143	0.80	
**4FA154[2]**	261	224	0.86	**0.83 ± 0.06**
	263	206	0.78	
	413	353	0.85	
	331	273	0.82	
**4FA154[2] + ΔCtpB**	271	159	0.58	**0.55 ± 0.08**
	227	113	0.50	
	280	170	0.61	
	287	148	0.52	
**4FA*[3]**	140	82	0.58	**0.63 ± 0.09**
	140	62	0.45	
	154	153	0.99	
	186	93	0.50	

[1] colony forming unit

[2] strain harboring only the C-terminal 4B cleavage site

[3] all 4B cleavage sites are deleted

Table S4. Plasmids Used in This Study, Related to the Experimental Procedures

Plasmid	Insert	Vector	Primer	Template	Reference
pDR211	Δss-ctpB-his$_6$	pET24a			(Campo and Rudner, 2006)
pDR212	Δss-ctpB^{S309A}-his$_6$	pET24a			(Campo and Rudner, 2006)
pKM35	Δss-4B-his6	pET28a			(Campo and Rudner, 2006)
pDT73	His$_6$-4FA$_{EC}$				(Campo and Rudner, 2006)
pRK02	ΔT86-ctpB- his$_6$	pET21b	T86 5' Nco + CtpB_Sal	pDR211	This work
pRK11	ΔV100-4B- his$_6$	pET21d	V100_Nco + 4B_Xho	pKM35	This work
pRK12	ΔQ43-ctpB- his$_6$	pET21b	Q43_Nde + CtpB_Sal	pDR211	This work
pRK13	ΔQ43-ctpB^{S309A}-his$_6$	pET21b	Q43_Nde + CtpB_Sal	pDR212	This work
pRK23	ΔQ43-ctpB-ΔPDZ-his$_6$	pET21b	Q43_Nde/Δ111-198 3’ + Δ111-198 5’/CtpB_Sal	pRK12	This work
pRK28	ctpB-PDZ-his$_6$	pET21b	PDZ_Nde + PDZ_Xho	pRK12	This work
pRK40	ctpB-R168A-his$_6$	pET21b	PDZ_R168A	pRK12	This work
pRK42	ctpB-R168F-his$_6$	pET21b	PDZ_R168F	pRK12	This work
pRK48	ctpB-Q338E-his$_6$	pET21b	CtpB_Q338E	pRK12	This work
pMM42	SUMO-4FA$_{151APA}$	pET SUMO	4FA 5' Nco + 4FA_APA	pRK85	This work
pMM43	SUMO-4FA$_{151AYV}$	pET SUMO	4FA 5' Nco + 4FA_APA_AYV	pRK85	This work
pMM44	SUMO-4FA$_{151DSE}$	pET SUMO	4FA 5' Nco +4FA_APA_DSE	pRK85	This work
pRK66	ctpB-V118Y-his$_6$	pET21b	PDZ_V118Y	pRK12	This work
pRK85	SUMO-4FA$_{EC}$[1]	pET SUMO	4FA 5' Nco + 4FA_Xho263, 4FA104A-E, 4FA116A-E, 4FA146V-E	pDT73	This work
pNC90A	amyE::spo4FA$^{A106R, S117R, V146F, G147Q, S155R}$				(Campo and Rudner, 2006)
pNC90A-155S	amyE::spo4FA$^{A106R, S117R, V146F, G147Q}$		4FA155S.F	pNC90A	This work
pRK100	ctpB-R168A/V118Y-his$_6$	pET21b	PDZ_V118Y	pRK40	This work
pRK101	ctpB-R168F/V118Y-his$_6$	pET21b	PDZ_V118Y	pRK42	This work

Table S5. Nucleotide Primer Used in This Study, Related to the Experimental Procedures

**Primer**	**Sequence**	**Usage**
V100_Nco	GATACCATGGTTCTTCCTGATTTAAAAGTT	PCR
4B_Xho	GATCTCGAGGCTTGCTTTTTCTTTTCCATA	PCR
Q43_Nde	GATGATCATATGCAAGCTGACTCTGAACGG	PCR
T86 5' Nco	GATACCATGGGAACGCTAAATGATCCTTATTC	PCR
CtpB_Sal	AGATAGTCGACATTGACAAATAATGATTTCAA	PCR
Δ111-198 5’	GATTCCTCACTCGAAACGGTTTTTGCA	PCR
Δ111-198 3’	CGTTTCGAGTGAGGAATCAAGAGAATC	PCR
PDZ_Nde	GATGATCATATGTCATTTGAAGGCATCGG	PCR
PDZ_Xho	GATCTCGAGCGGAATCTCAGCTCT	PCR
PDZ_R168A	CACGCTGTGTTAAAAATAGCAGGAAAAAAAGGGTCCAGC	Mutation: CtpBR168A
PDZ_R168F	CACGCTGTGTTAAAAATATTCGGAAAAAAAGGGTCCAGC	Mutation: CtpBR168F
PDZ_V118Y	GAAGGCATCGGGGCTGAGTACGGAATGGAAGACGGCAAA	Mutation: CtpBV118Y
CtpB_Q338E	GGAAAGGGAACGGTTGAACAGGCTGTGCCAATG	Mutation: CtpBQ338E
4FA_APA	GATCTCGAGTTATGCAGGCGCGATCAGATCTTTGCCTAC	PCR
4FA_APA_AYV	GATCTCGAGTTACACATACGCGATCAGATCTTTGCCTAC	PCR
4FA_APA_DSE	GATCTCGAGTTATTCAGAATCGATCAGATCTTTGCCTAC	PCR
4FA 5' Nco	GATGCCATGGATTATAAAACAAACATTGGA	PCR
4FA Xho263	GATCTCGAGTTATTCAAATGAAATCAC	PCR
4FA104A-E	CAGTCAGATTAAACCCGAGGTAGCCAAAACCTTTG	Mutation: $4FA^{A104E}$
4FA116A-E	ACTGAATTTCAATTTGAGTCAGCAAGCCATTGG	Mutation: $4FA^{A116E}$
4FA146V-E	GAACAGCAGATTGAAGAAGGCAAAGATCTGATC	Mutation: $4FA^{V146E}$
4FA155S.F	GATCGCGCCTGCATCCGGGAAAGTACAGC	Mutation: $4FA^{155S}$ (back to wt)

Table S6. *B. subtilis* Strains Used in the Study, Related to the Experimental Procedures

Strain	Genotype	Reference
BNC243	*spoIVFΔAB::cat, amyE::spoIVFA (spec), lacA::P$_{spoIVA}$ ATG spoIVFB (erm)*	(Campo and Rudner, 2006)
BNC694	*spoIVB::phleo, spoIVF::cat, lacA::PspoIVF-B (erm), amyE::PspoIVF-gfp-A (spec)*	(Campo and Rudner, 2006)
BNC734	*spoIVF::cat, lacA::PspoIVF-B (erm), amyE::PspoIVF-A (spec), ctpB::tet*	(Campo and Rudner, 2006)
BNC850	*spoIVFΔAB::cat, amyE::spoIVFA$^{A106R, S117R, V146F, G147Q, S155R}$, (spec), lacA::P$_{spoIVA}$-ATG-spoIVFB (erm)*)	(Campo and Rudner, 2006)
BDR2601	*spoIVFΔAB::cat, amyE::spoIVFA$^{A106R, S117R, V146F, G147Q}$, (spec), lacA::P$_{spoIVA}$-ATG-spoIVFB (erm)*	This work
BDR2603	*spoIVFΔAB::cat, amyE::spIVFA$^{A106R, S117R, V146F, G147Q}$, (spec), lacA::P$_{spoIVA}$-ATG-spoIVFB (erm), ctpB::tet*	This work
