# Supplementary material for: CtpB Assembles a Gated Protease Tunnel Regulating Cell-Cell Signaling during Spore Formation in Bacillus subtilis
Source: Cell. 2013 Oct 24;155(3):647–58. doi: 10.1016/j.cell.2013.09.050 (PMC3808539; doi:10.1016/j.cell.2013.09.050)
Supplement: Document S2. Article plus Supplemental Information [file mmc2.pdf]

CtpB Assembles a Gated Protease Tunnel Regulating Cell-Cell Signaling during Spore Formation in *Bacillus subtilis*

Markus Mastny,[1,6] Alexander Heuck,[1,6] Robert Kurzbauer,[1,6] Anja Heiduk,[2] Prisca Boisguerin,[2,7] Rudolf Volkmer,[2] Michael Ehrmann,[3,4] Christopher D.A. Rodrigues,[5] David Z. Rudner,[5] and Tim Clausen[1,*]
[1]Research Institute of Molecular Pathology, 1030 Vienna, Austria
[2]Institute of Medical Immunology, Charité, Universitätsmedizin Berlin, 13353 Berlin, Germany
[3]Center for Medical Biotechnology, University of Duisburg-Essen, 45117 Essen, Germany
[4]School of Biosciences, Cardiff University, Cardiff CF10 3US, UK
[5]Department of Microbiology and Immunobiology, Harvard Medical School, Boston, MA 02115, USA
[6]These authors contributed equally to this work
[7]Present address: Centre de Recherches de Biochimie Macromoléculaire, UMR 5237 CNRS, 34293 Montpellier 5, France
*Correspondence: clausen@imp.univie.ac.at
http://dx.doi.org/10.1016/j.cell.2013.09.050

SUMMARY

Spore formation in *Bacillus subtilis* relies on a regulated intramembrane proteolysis (RIP) pathway that synchronizes mother-cell and forespore development. To address the molecular basis of this SpoIV transmembrane signaling, we carried out a structure-function analysis of the activating protease CtpB. Crystal structures reflecting distinct functional states show that CtpB constitutes a ring-like protein scaffold penetrated by two narrow tunnels. Access to the proteolytic sites sequestered within these tunnels is controlled by PDZ domains that rearrange upon substrate binding. Accordingly, CtpB resembles a minimal version of a self-compartmentalizing protease regulated by a unique allosteric mechanism. Moreover, biochemical analysis of the PDZ-gated channel combined with sporulation assays reveal that activation of the SpoIV RIP pathway is induced by the concerted activity of CtpB and a second signaling protease, SpoIVB. This proteolytic mechanism is of broad relevance for cell-cell communication, illustrating how distinct signaling pathways can be integrated into a single RIP module.

INTRODUCTION

Cellular differentiation is a process commonly associated with multicellular organisms and their development. However, differentiation into specialized cell types is also employed by a range of bacteria. One classic example is the endospore forming bacterium *Bacillus subtilis* that can differentiate into dormant and stress-resistant spores to survive harsh environmental conditions (Setlow, 1995). Spore formation proceeds via an asymmetric cell division resulting in a small cell, the forespore, and a large mother cell (Errington, 2003; Stragier and Losick, 1996). The two cells follow distinct developmental programs that are kept in register with each other by a tightly coordinated intercellular signaling casade. The corresponding "criss-cross" communication is mediated by the sequential activation of cell-type-specific sigma factors termed σ^E, σ^F, σ^G, and σ^K (Kroos et al., 1989; Losick and Stragier, 1992) (Figure 1A). The final steps of spore formation are under control of the sigma factor σ^K, which is synthesized in the mother cell as an inactive, membrane-associated precursor protein (pro-σ^K) that is activated by regulated intramembrane proteolysis (RIP) (Wolfe and Kopan, 2004). In the specialized SpoIV RIP pathway, the I-CLiP intramembrane cleaving protease SpoIVFB (4FB) is embedded in the mother-cell membrane and is held inactive by two membrane proteins SpoIVFA (4FA) and BofA (Cutting et al., 1990; Resnekov and Losick, 1998; Rudner et al., 1999; Rudner and Losick, 2002). Inhibition of 4FB is relieved by the signaling proteases SpoIVB (4B) and CtpB that are secreted into the intramembrane space and cleave the extracellular domain of the negative regulator 4FA at multiple sites (Campo and Rudner, 2006; Zhou and Kroos, 2005). The 4FB membrane protease then processes pro-σ^K into the mature σ^K transcription factor that, in turn, activates many of the genes required to complete the sporulation program (Campo and Rudner, 2006, 2007; Pan et al., 2003). Though the members of this specialized RIP pathway are known, it is still unclear how the distinct proteases cooperate with each other to ensure the precisely timed activation of σ^K.

The signaling proteases 4B and CtpB belong to the widespread family of PDZ-proteases that combine a catalytic serine protease with a regulatory PDZ domain that is capable of sensing distinct molecular cues, as exemplified by the HtrA proteases (reviewed in Clausen et al., 2011). In contrast to HtrA proteases, the mechanistic features of 4B and CtpB have not been examined, and thus it remains unknown how these signaling proteases are regulated. Whereas 4B belongs to a small clan of bacterial serine proteases of unknown structure, CtpB is a member of the large family of C-terminal processing proteases

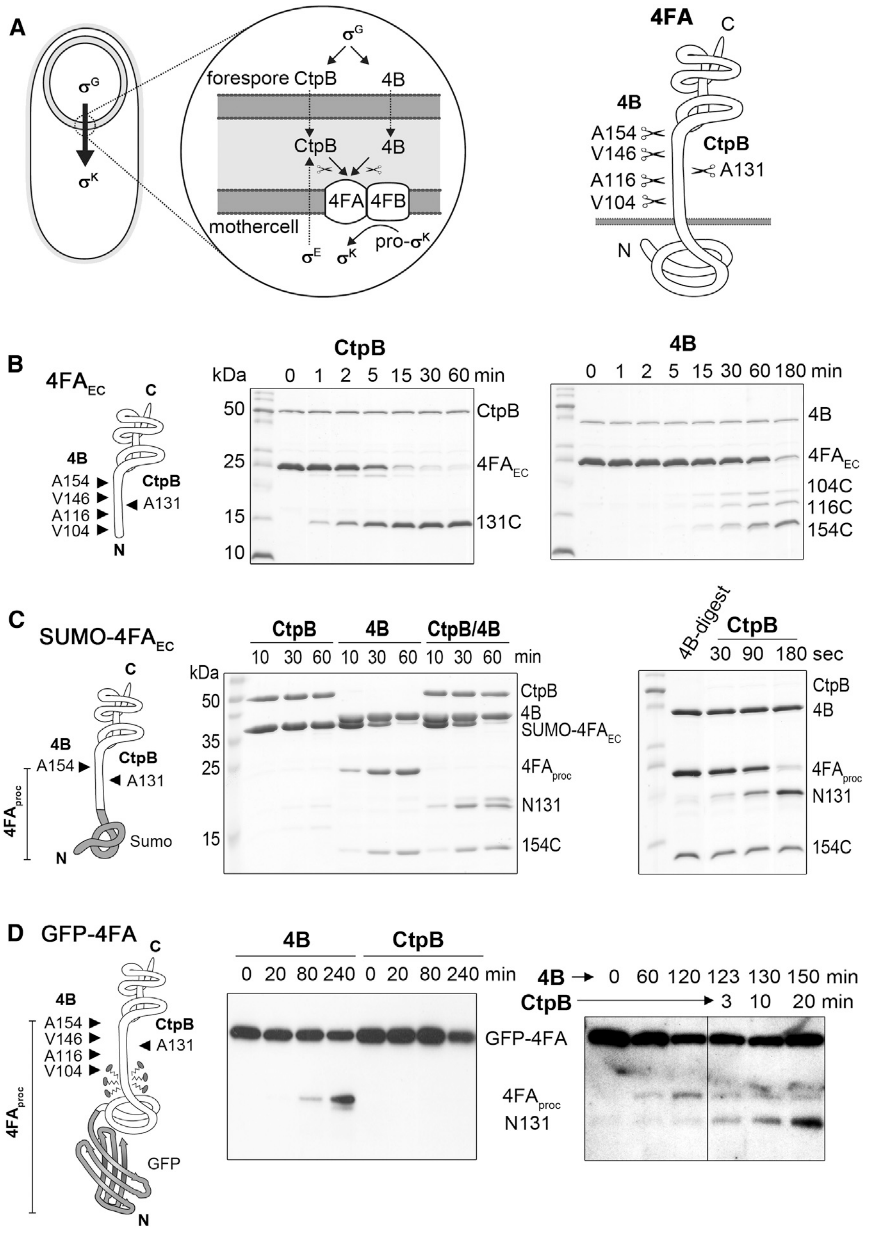

Figure 1. Sequential Processing of 4FA by 4B and CtpB

(A) Illustration of the SpoIV signaling pathway between forespore and mother cell during sporulation. The 4FA regulatory protein (highlighted) is composed of a globular N-terminal domain, a transmembrane helix (residues 73-90), an unstructured linker region and a compact C-terminal domain (residues 160–255, LytM-like) extending into the intermembrane space. Cleavage sites targeted by the signaling proteases 4B and CtpB are indicated. For simplicity, a second regulatory protein (BofA) is not shown.

(B) Cleavage of the 4FA extracellular portion ($4FA_{EC}$, shown schematically on the left with corresponding cleavage sites and products) as monitored by SDS-PAGE analysis.

(C) Cleavage of the SUMO-$4FA_{EC}$ substrate having only one 4B cleavage site. Right: illustration of the rapid degradation of the 4B-processed substrate $4FA_{proc}$ by CtpB, which was applied in 10-fold dilution compared to the $4FA_{EC}$ cleavage assay.

(D) Detergent-solubilized GFP-4FA was cleaved by 4B but not by CtpB. However, CtpB was able to rapidly cleave the 4B preprocessed substrate $4FA_{proc}$ (right). A previous in vivo analysis indicates that GFP-4FA is cleaved by 4B at position 154 (Campo and Rudner, 2006). See also Figure S1.

(Rawlings et al., 2002) that either serve a regulatory role when cutting the C-termini of specific precursor proteins (Hara et al., 1991; Takahashi et al., 1988) or a housekeeping function when degrading incompletely synthesized proteins tagged by the SsrA system (Keiler et al., 1996). Structural analysis of the chloroplast D1 degrading protease (D1P) (Liao et al., 2000), a member of this protease family involved in the biogenesis of photosystem II, revealed a Ser-Lys dyad as the catalytic motif but did not elucidate further regulatory properties.

Given the remarkable versatility of PDZ-proteases acting as sensor proteins in diverse biological processes and the broad impact of RIP cascades for cell-cell communication, it is striking how little is known about the molecular mechanism of C-terminal processing proteases implicated in cellular signaling. To delineate this mechanism, we undertook a comprehensive structural, biochemical, and in vivo analysis of CtpB from *B. subtilis*. Here, we present high-resolution crystal structures of CtpB in a resting and multiple active conformations. A prominent feature of the CtpB fold is the sequestration of the catalytic pocket in a narrow tunnel providing a mechanistic explanation for the observed dependency of CtpB protease activity on prior substrate processing. In sum, our data define the molecular underpinnings of how CtpB is regulated and explains how a specialized RIP system ensures the precise temporal control of transcription factor activation that is required for efficient spore formation.

RESULTS

The 4B Protease Prepares the 4FA Substrate for CtpB Cleavage

Previous studies revealed that σ^K maturation is under control of the signaling proteases 4B and CtpB that introduce several cuts into the extracellular domain of the 4FA inhibitor. The cleavage sites targeted by 4B (residues 104, 116, 146, and 154) and CtpB (residue 131) (Campo and Rudner, 2006) are located in the linker region connecting the compact LytM domain with the

transmembrane region of 4FA (Figure 1A). To test whether the two PDZ-proteases cleave the 4FA substrate independently or in concert, we incubated various 4FA constructs with recombinant 4B and CtpB. Owing to autoproteolysis of wild-type CtpB at positions 36 and 42, we performed the biochemical analysis using a truncated protease, which had an activity indistinguishable from the full-length protein (Figures S1A and S1B available online). Consistent with previous work (Campo and Rudner, 2006), the model substrate $4FA_{EC}$ that represents the complete extracellular portion of 4FA was efficiently cleaved by both 4B and CtpB (Figure 1B). Because the N terminus of the extracellular 4FA domain is normally anchored in the mother-cell membrane and thus shielded from degradation, we generated a 4FA variant carrying an N-terminal SUMO fusion protein as a steric block. To simplify our analysis, we further mutated all 4B cleavage sites except 154, the primary target site of 4B in vivo (Campo and Rudner, 2006). Strikingly, CtpB was unable to process the "SUMO-sealed" substrate, whereas 4B efficiently cut the construct into two halves. However, when SUMO-$4FA_{EC}$ was incubated with both signaling proteases, the N-terminal cleavage product ($4FA_{proc}$) generated by 4B was rapidly cleaved by CtpB (Figure 1C). This CtpB-dependent cleavage of the preprocessed substrate was >10-fold faster than of the uncut, N-terminally accessible $4FA_{EC}$ substrate.

To monitor the activities of the proteases with a substrate that more closely mimics the in vivo situation, we prepared membranes from early sporulating *B. subtilis* cultures. The strain used (BNC694) carries a deletion of the 4B protease and expresses a functional 4FA transmembrane variant with an N-terminal GFP fusion. The detergent-solubilized *B. subtilis* membrane proteins were incubated with CtpB and 4B alone, and the cleavage of 4FA was followed by immunoblot analysis using anti-GFP antibodies. Consistent with our previous results, solubilized GFP-4FA was cleaved by 4B, but not by CtpB (Figure 1D). Moreover, treatment with 4B yielded a single cleavage product, resulting from a cut at position 154, even though all 4B cleavage sites were present. This suggests that the 4B protease cleaves the full-length, membrane-embedded substrate differently from the isolated 4FA extracellular domain. Most importantly, the corresponding cleavage product was promptly degraded by CtpB, which was added at a later time point to the reaction (Figure 1D). Taken together, the in vitro reconstituted cleavage reactions demonstrate that 4B-dependent cleavage of 4FA generates a substrate that can be further digested by CtpB.

The CtpB Protease Is Organized as a Dimeric Ring

To further investigate the molecular mechanism underlying the interplay between the two signaling proteases, we performed a detailed structural analysis of *B. subtilis* CtpB. The crystal structure of the wild-type protease was determined at 1.9 Å resolution using anomalous diffraction data (Table S1). Overall, CtpB is composed of a PDZ domain, two protease subdomains, and two dimerization motifs, which are used to assemble a ring-like dimer (Figures 2 and S2A). The N-terminal dimerization motif comprises two alpha helices (α1, α2) that form an intermolecular 4-helix bundle upon dimerization. On the opposite side of the CtpB dimer, the C-terminal dimerization motifs folds into two juxtaposed 4-helix domains (α9, α10, α11, α12) that resemble, despite distinct function, the pro-MMP-2 propeptide (Morgunova et al., 1999) and the peptidoglycan-binding domain of the lytic transglycosylase gp144 (Fokine et al., 2008). Consistent with the large size of the composite dimer interface (2550 $Å^2$), its hydrophobic character, and the participation of highly conserved residues (Figure S2B), the CtpB dimer observed in the crystal is stably formed in solution and is essential for protease function (Figures S1C and S1D).

The PDZ domain that protrudes away from the center of the dimer is the most flexible entity of the protein as judged by its thermal motion factors (46.3 $Å^2$ compared to the average B-factor of 41.5 $Å^2$). It adopts a similar fold as other PDZ domains present in a protease context having a highly twisted, five-stranded β sheet (βc, βd, βe, βf, and βg) as the central element. The edges of the sheet are shielded by two helices, α4 and α5, with the longer helix α5 contributing to the peptide-binding cleft next to strand βc. The functional body of CtpB is completed by a bipartite protease scaffold that is composed of a cap and a core subdomain (Figure 2C). The protease cap forms a roof-like structure on top of the protease core, with one side of the roof being formed by helix α3 and the second side by the 3-stranded sheet βa/βn/βo. The protease core comprises a 4-stranded, largely parallel β sheet (βh, βi, βj, and βm) that winds around helix α13 and is covered on its opposite side by helices α6, α7, and α8. Compared to other C-terminal processing proteases (peptidase family S41) (Rawlings et al., 2002), CtpB features a 27-residue long insertion after helix α7 that folds into a β-hairpin structure (βk and βl). This insertion protrudes away from the protease core and continues along the cap domain toward the N-terminal dimerization domain of the adjacent protomer, where Arg280 located at the tip of the βk/βl-hairpin closely interacts with helix α2 thus contributing to the CtpB dimer interface (Figure S2A).

The Proteolytic Site of CtpB Is Buried within a Tunnel

The most remarkable feature of the CtpB structure is a narrow tunnel that is formed between the protease core and the protease cap (Figure 3A). This tunnel is about 7 Å wide and therefore should exclusively allow the passage of an unfolded protein segment present in extended conformation. The tunnel opens toward the peptide-binding cleft of the PDZ domain and sequesters the active site nucleophile (Ser309) required for peptide cleavage (Campo and Rudner, 2006). The side chain of Ser309 is in hydrogen bonding distance to Lys334, which could function as an acid-base catalyst during peptide hydrolysis. Lys334, in turn, is oriented by Gln338, a strictly conserved residue in the CtpB family (Figure 3B and Figure S2B). Owing to the observed interaction network, we presume that Ser309, Lys334, and Gln338 function as catalytic triad mediating cleavage of the 4FA substrate. Notably, a peptide ligand, which copurified and cocrystallized with CtpB, was observed at the entrance of the protease tunnel near the catalytic triad (Figure 3B). The peptide closely interacts with strand βn revealing how the P2, P3, and P4 residues of the substrate are accommodated in specificity pockets formed at the interface of the two protease subdomains.

The PDZ Domain Controls Access to the Protease Tunnel

As will be described below, we determined crystal structures of various functional forms of CtpB that recapitulate the enzymatic

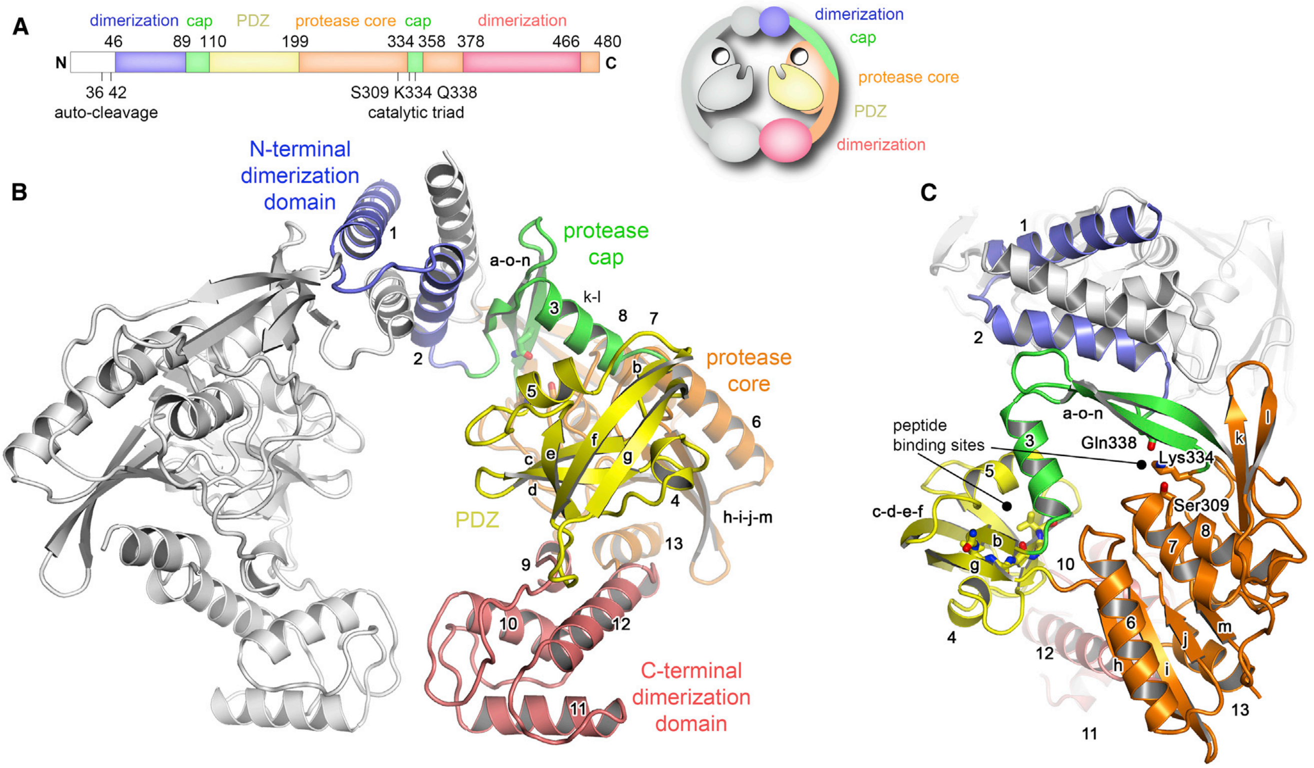

Figure 2. Structural Architecture of the CtpB Dimer

(A) Domain organization of CtpB indicating the domain borders, autocleavage sites and catalytic residues.

(B) The ribbon model of the CtpB dimer shows one CtpB protomer in white, while the second protomer is colored according to its domains with the secondary structural elements being labeled. The schematic drawing illustrates the overall organization of the CtpB dimer.

(C) Orthogonal view of the CtpB dimer highlighting the tunnel formed between protease cap and protease core. Residues constituting the proteolytic site (Ser309, Lys334, Gln338) and the carboxylate-binding loop of the PDZ domain (Gly113–Ala116) are shown as stick models. See also Figures S1 and S2 and Table S1.

cycle of the protease. Based on the proper arrangement of the catalytic triad, the structure described above likely reflects the active state of CtpB, which we refer to as CtpBa1. To refine the molecular mechanism of CtpB regulation, we carried out additional crystallization experiments and obtained a crystal form in which CtpB was present in two further active conformations, CtpBa2 and CtpBa3 (Table S1). In contrast to CtpBa1, the peptide bound in the proteolytic site of CtpBa2/3 was covalently attached to Ser309 representing a trapped reaction intermediate that depicts the mechanistic properties of CtpB in great detail (Figure S3A). The organization of the active site suggests that binding specificity primarily depends on the P1 and P2 residues preceding the scissile bond. The P1 side chain of the substrate is accommodated in a small, hydrophobic pocket lined with residues Leu257, Ile313, and Val337, whereas the P2 residue protrudes into a slightly larger hydrophobic pocket that is composed of residues from both protease core (Tyr256) and cap (Phe103 and Leu107). Consistent with the structural data, the biochemically identified CtpB cleavage sites have specific residues in P1 (Ala and Val) and P2 (Thr, Leu, and Ile) position but are otherwise diverse (Figure S3B). In addition, structural comparison of CtpBa1/2/3 reveals that protease and dimerization domains are similarly arranged (rms deviation between 0.50–0.59 Å for 300 Cα atoms), whereas the PDZ domains are differently positioned. In CtpBa2 and CtpBa3, the PDZ domains are located further from the proteolytic tunnel compared to CtpBa1, highlighting the pronounced en-bloc mobility of the PDZ domain (Figure 3C). Despite this structural flexibility, a common feature of the active protease is the formation of the two-stranded β sheet βb/βg that limits the rotational freedom of the PDZ domain to orient its peptide-binding site toward the opening of the protease tunnel.

To fully delineate how CtpB is regulated, we determined the high-resolution crystal structure of the resting protease by crystallizing the catalytically inactive S309A mutant. Compared to CtpBa1/2/3, the resting CtpB (CtpBi) assembles a more compact dimer with a remodeled functional core. Interestingly, the PDZ domain was repositioned such that it is closely incorporated into the CtpB scaffold blocking the protease tunnel (Figure 3C). As a further consequence, the protease cap is shifted away from the protease core leading to the remodeling of the active site. This rearrangement is best seen for Gln338 that pulls the active site Lys334 away from Ser309, thereby disrupting the catalytic triad (Figure S3C). Accordingly, the resting CtpBi is characterized by a disrupted active site and a blocked enzymatic tunnel. Taken together, the four crystal structures of CtpB represent distinct snapshots of the working CtpB emphasizing the importance of the PDZ domain in

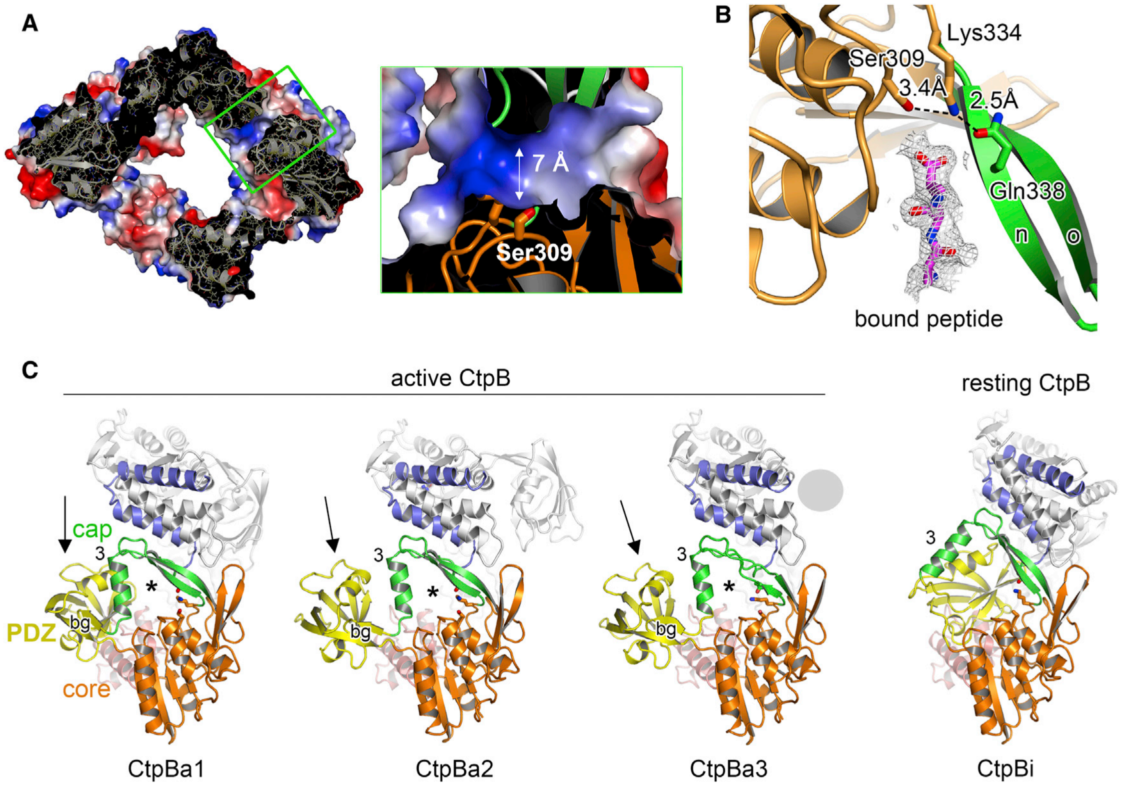

Figure 3. The Proteolytic Site of CtpB Is Sequestered in a Narrow Tunnel

(A) Surface presentation of the CtpB dimer with mapped electrostatic potential. The molecule is shown in a half-cut view displaying the construction of one of the two protease tunnels penetrating the dimer. The zoomed-in window illustrates the position of the catalytic serine buried within the tunnel, as well as the dimensions of the molecular passage.

(B) The catalytic residues Ser309, Lys334, and Gln338 are shown in stick representation and their respective distances are indicated. The omit electron density of a cocrystallized ligand (1.9 Å resolution, contoured at 1.2 σ) is shown together with a modeled poly-Ala peptide.

(C) Ribbon plots of CtpBa1, CtpBa2, CtpBa3, and CtpBi. To illustrate the pronounced rearrangements of the protease cap, helix α3 is labeled. Moreover, the variable positions of the PDZ domain are indicated by an arrow, the opening of the proteolytic tunnel is marked with an asterisk and the β sheet b-g, which was only observed in the active conformations, is labeled. In CtpBa3, one PDZ domain was too flexible to be modeled into electron density and is indicated by a gray sphere. See also Figure S3.

gatekeeping the protease tunnel and defining resting and active conformations.

Peptide Binding to the PDZ Domain Stimulates Protease Activity

Having observed different functional conformations of CtpB, we next explored the molecular mechanism underlying the regulatory switch in protease activity. Strikingly, inspection of the ligand-binding sites of the four CtpB structures revealed that the active structures CtpBa1/2/3 had a peptide bound in the PDZ domain (Figure 4A), whereas the corresponding interaction site of CtpBi was empty. The peptide-binding pocket of the PDZ domain is located in the cleft between strand βc and helix α5. One edge of this pocket is composed of the carboxylate-binding loop, which is formed by residues Gly113-Ile114-Gly115-Ala116 (Figure 4B) and anchors the C terminus of the captured substrate. Although we could not determine the identity of the copurified and cocrystallized peptide by mass spectrometry, the high quality of the CtpBa1 electron density map revealed a proline as the penultimate residue, which is bound together with its adjacent residues as an additional β strand to βc of the PDZ domain (Figure 4A). Since proline residues are typically not found in PDZ-binding motifs, we explored the substrate preference of the PDZ domain of CtpB by biochemical means. To this end, we carried out a peptide spot analysis using a polypeptide library presenting 1,849 distinct C termini prepared by an inverted peptide array (Boisguerin et al., 2007) (Figure S4A). Peptides recognized by the isolated PDZ domain of CtpB were systematically permutated and tested in secondary screens. The results of these assays indicate that the PDZ domain of CtpB preferentially binds peptides carrying Tyr/Phe/Trp-Ala/Val combinations at their C terminus (Y/F/W-V/A, Figures 4C and S4B). To test whether the identified ligands also interact with full-length CtpB, we carried out isothermal titration calorimetry (ITC) measurements with the proteolytically inactive S309A mutant. The derived dissociation constants (K_D) for the full-length protein were about 3-fold higher than for the isolated PDZ domain, but still in the range of 2–10 μM highlighting the potential of the Y/F/W-V/A motif to target substrates to CtpB (Figure S4C). However, when we analyzed the sequences of the 1,864 proteins predicted to reside

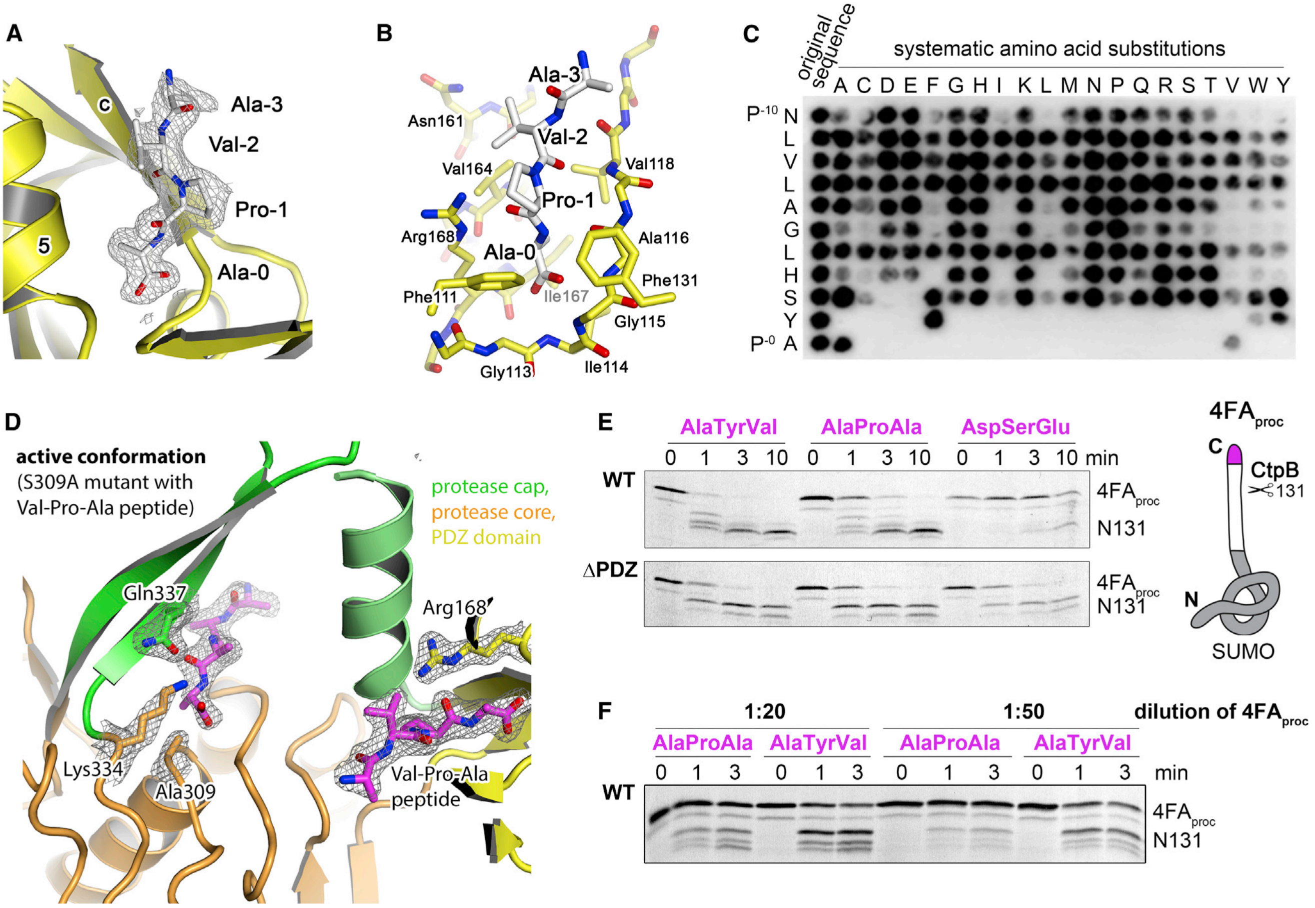

Figure 4. Peptide Binding to the PDZ Domain Regulates CtpB Activity

(A) Omit electron density of a cocrystallized peptide bound to the PDZ domain (1.9 Å resolution, contoured at 1.2 σ).

(B) Detailed presentation of the substrate-binding pocket of the PDZ domain with bound peptide ligand (white). Residues involved in peptide binding are shown in stick presentation and are labeled.

(C) Secondary peptide spot screen of an identified PDZ binder (NH_2-NLVLAGLHSYA-COOH, spot 594 in Figure S4A). As indicated, amino acids were systematically varied.

(D) Ribbon plot of the proteolytically impaired S309A (colored according to domains) that was cocrystallized with the NH_2-EMDKPQTAAVPA-COOH peptide in the active conformation. Peptides bound to the proteolytic site and PDZ domain (stereoview in Figure S4D) and selected CtpB residues are shown in stick mode and are overlaid with the corresponding 2Fo-Fc omit electron density (1.9 Å resolution, contoured at 1.2 σ).

(E) Cleavage assays of in vitro translated $4FA_{proc}$ constructs carrying distinct C termini. Substrates and products are visualized by ^{35}S-methionine autoradiography and are labeled.

(F) Cleavage assays of $4FA_{proc}$ constructs that were applied at decreasing concentrations. See also Figure S4 and Table S2.

in the intermembrane space of *B. subtilis*, none of the 13 proteins carrying the preferred C-terminal consensus motif appears to be a potential substrate. In all cases, the Y/F/W-V/A C terminus is close to a folded protein segment and thus presumably inaccessible to CtpB (Table S2). Given the high affinity of the Y/F/W-V/A motif, it is striking that a peptide with a proline in penultimate position, which was a minor hit in the peptide spot analysis, copurifies with CtpB and stabilizes its active fold. As this peptide was only observed upon overexpressing proteolytically active CtpB, we presume that the copurified ligand was directly generated by CtpB, most likely at rather high concentrations favoring its binding to the PDZ domain. Unfortunately, we could not determine the K_D of a corresponding peptide due to its weak binding properties. To investigate the interaction with a proline-containing peptide, we thus applied a structural approach and cocrystallized the S309A mutant with an oligopeptide having a Val-Pro-Ala C terminus. The crystal structure of the resultant S309A/VPA complex, which was determined at 1.9 Å resolution, demonstrated unequivocally that the Val-Pro-Ala motif can specifically bind to the PDZ domain (Figure S4D). In addition, the S309A/VPA structure revealed that peptide binding itself is sufficient to transform the resting protease into the active conformation, even in the absence of the functional OH group of Ser309 (Figure 4D).

To test the respective allosteric mechanism biochemically, we carried out degradation assays monitoring how CtpB digests model substrates carrying distinct C termini. Based on the $4FA_{proc}$ substrate resulting from 4B cleavage, we synthesized various model proteins containing the native CtpB cleavage

site and variable C termini predicted to having either a high-affinity (Ala-Tyr-Val), a low-affinity (Ala-Pro-Ala, reflecting the 4B-generated, physiological substrate), or a noncognate (Asp-Ser-Glu) PDZ-targeting tag. Upon incubation with CtpB, we monitored the degradation of the in vitro translated proteins by autoradiography. In support of our hypothesis that peptide binding is coupled to protease activation, substrates carrying the Ala-Tyr-Val or Ala-Pro-Ala C terminus were rapidly degraded, while the noncognate C terminus strongly inhibited degradation by CtpB (Figure 4E). Moreover, the low-affinity targeting tag Ala-Pro-Ala was less efficient in stimulating protease function than the Ala-Tyr-Val motif, especially at lower concentrations (Figure 4F), although the difference was smaller than would be expected from its markedly lower binding affinity.

Arg168 of the PDZ Domain Couples Substrate Binding with Protease Activation

Owing to its residual protease activity against substrates having a noncognate C terminus (Figures 1B and 4E), CtpB appears to exist in equilibrium between resting and active conformations. Of note, the PDZ domain of CtpBi is fixed in its inhibitory position by a multitude of specific interactions with residues of the protease cap, the protease core, and the tail domain (Figure S5A). The size of this interface (1300 $Å^2$) corresponds to interface areas observed for transient and stable protein-protein interactions (i.e., 500–1500 $Å^2$) and, importantly, is larger than that observed in the active conformations (e.g., 550 $Å^2$ in CtpBa1). Therefore, the resting state of CtpB with its "locked" PDZ domain should represent the prevailing conformation in solution. Given this favored domain arrangement, how can the catalytically active conformation be stabilized? Our structural data suggest that Arg168 could function as a key residue controlling the conformational switch (Figure 5A). In the resting state, the side chain of Arg168 points away from the PDZ domain forming hydrogen bonds with Ser106 that is located on helix α3 of the protease cap. However, in the activated state, Arg168 is reoriented such that its side chain can directly participate in anchoring the main-chain of the incoming ligand (Figure 5B). Importantly, Arg168 maintains the contact with helix α3 during the conformational transition, as seen by its interactions with Ser106 or Asp105 in CtpBa1 and CtpBa2/3, respectively. Thus, Arg168 can switch between peptide bound and unbound orientations but maintains the interdomain contact in both states. We therefore propose that Arg168 functions as a substrate sensor stabilizing the active conformation once it is engaged in peptide binding to the PDZ domain (Figure 5B).

To test this model, we monitored the effects of various CtpB mutations on degrading the $4FA_{proc}$ substrate (Figure 5C). Since Arg168 closely interacts with the protease cap orienting the flexible domain, we first explored to what extent the precise positioning of the protease cap is required for full protease function. For this purpose, we distorted the catalytic triad Ser309-Lys334-Gln338, which contain residues from both protease subdomains. Introducing the Q338E mutation abolished protease function confirming the identity of the composite catalytic triad. Moreover, this result argues that the cap needs to be precisely oriented to establish the fully functional protease. Next, we exchanged Arg168 by Ala and Phe thereby distorting the interface between PDZ domain and protease cap to different degrees. Consistent with the proposed model, the introduced R168A and R168F mutations had two effects. On one side, the mutations led to a 3- to 5-fold weaker affinity for PDZ ligands confirming the involvement of Arg168 in binding the C terminus of the incoming substrate (Figure S5B). In parallel, the mutations reduced the proteolytic activity of CtpB against $4FA_{proc}$ with the R168A mutation having a milder effect than the R168F exchange (Figure 5C). To specifically monitor the catalytic function of the Arg168 mutants, we generated double mutants coupling the Arg168 variants with a V118Y mutation that completely blocks peptide binding to the PDZ domain (Figure S5) but still exhibits residual protease activity (Figure 5C). Importantly, the combined R168A/V118Y and R168F/V118Y mutations reduced the protease activity of CtpB to levels lower than the V118Y mutation alone. These results highlight the additional catalytic function of Arg168. To better understand this catalytic role, we crystallized the R168A mutant in the active conformation. As illustrated in Figure S5D, the functional cap and PDZ domains are arranged similarly as in wild-type CtpB; however, both domains displayed elevated thermal motion factors. In particular, helices α3 and α5, where the interacting Ser106 and Arg168 residues protrude, became more flexible and were slightly relocated. These data indicate that the physical link between PDZ and protease domain, which is established by Arg168, is critical to assemble the fully functional CtpB protease. At last, to directly test the filtering function of the PDZ domain, we monitored protease activity and substrate selectivity of a PDZ deletion mutant (ΔPDZ). Strikingly, the ΔPDZ mutant cleaved the $4FA_{proc}$ substrate more efficiently than wild-type CtpB (Figure 5C). Moreover, the ΔPDZ mutant did not distinguish between substrates having a CtpB-targeting or nontargeting C terminus, as it degraded all substrates with similar efficiency (Figure 4E). Thus, the PDZ deletion yields a constitutively active, "open gate" protease. Taken together, these data demonstrate that the repositioning of the PDZ domain upon substrate binding is critical for protease regulation and is communicated via Arg168 to the proteolytic site.

4B/CtpB-Mediated Removal of 4FA Residues 131–154 Induces σ^K Maturation

To explore the cooperation of 4B and CtpB in degrading the RIP inhibitor 4FA in vivo, we followed the processing of σ^K in different *B. subtilis* strains. For this purpose, bacterial lysates prepared in 30 min intervals after inducing sporulation were subjected to immunoblot analysis. Consistent with previous reports (Campo and Rudner, 2006; Cutting et al., 1990; Pan et al., 2003), the 4FB-mediated processing of pro-σ^K into σ^K started in the wild-type strain about 3.5 hr after inducing sporulation, whereas mutating all 4B cleavage sites delayed pro-σ^K processing and consequently reduced spore formation (Figure 6). Moreover, a CtpB deletion strain showed a delay of 30-60 min in the onset of σ^K processing. A similar "delay" phenotype was observed in the strain expressing the $4FA^{154}$ variant carrying only the most C-terminal 4B cleavage site (Ala154), the primary target site of 4B in vivo (Campo and Rudner, 2006). To test our model that 4B and CtpB cooperatively degrade 4FA, we generated a combined "delay" strain expressing $4FA^{154}$ and lacking the CtpB

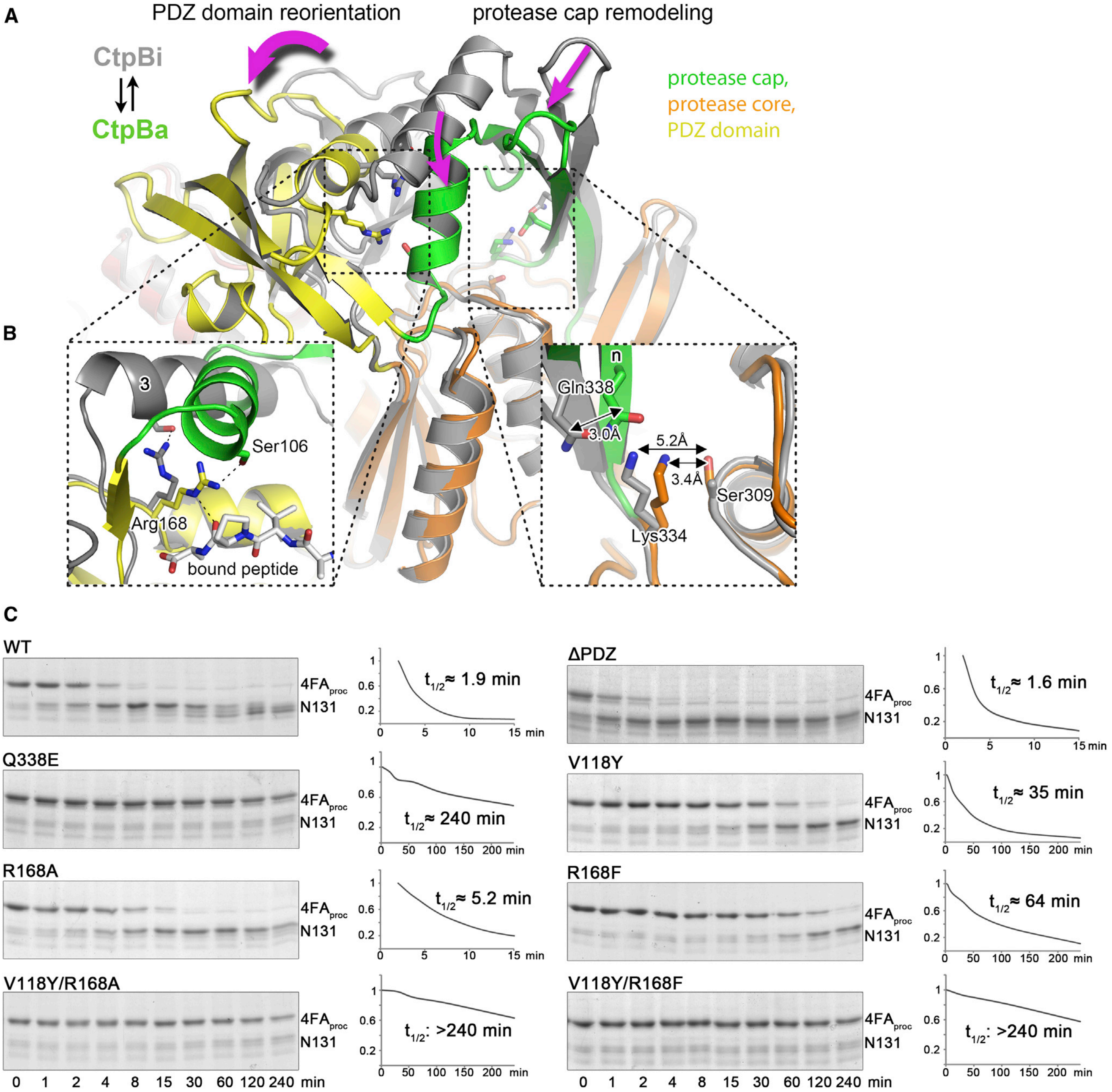

Figure 5. Peptide Binding to the PDZ Domain Stabilizes the Active State

(A) CtpBa1 (colored according to domains) and CtpBi (gray) are shown with the protease core domains superimposed. Arrows indicate movements of the protease cap and the PDZ domain that accompany the transition from the resting to the active conformation.

(B) Zoomed-in windows illustrate key events underlying the switch in activity. Left: Aligned peptide-binding sites of the PDZ domain highlighting the catalytic role of Arg168. Upon substrate binding, the reoriented Arg168 can undergo additional interactions with the captured ligand (white) thereby stabilizing the active protease form. Dotted lines indicate hydrogen bonds. Right: Remodeling of the proteolytic site upon rearrangement of the protease cap. Distances between the functional groups of the catalytic residues are indicated and reveal the remodeling of the catalytic triad during the conformational switch.

(C) SDS-PAGE assays monitoring the degradation of $4FA_{proc}$ by wild-type CtpB and various mutants (substrates and cleavage products are indicated). The right panels illustrate the time courses of substrate degradation yielding the indicated half-times of substrate cleavage. See also Figure S5.

protease. In agreement with our model, this strain exhibited a synthetic phenotype, as pro-σ^K processing was severely impaired. Moreover, spore formation of the ΔCtpB/$4FA^{154}$ double mutant was reduced to the same level as for the 4FA variant missing all 4B cleavage sites, whereas strains either lacking CtpB or expressing $4FA^{154}$ were almost indistinguishable from wild-type (Figure 6, Table S3). These findings provide evidence for the close cooperation between 4B and CtpB in vivo and, in

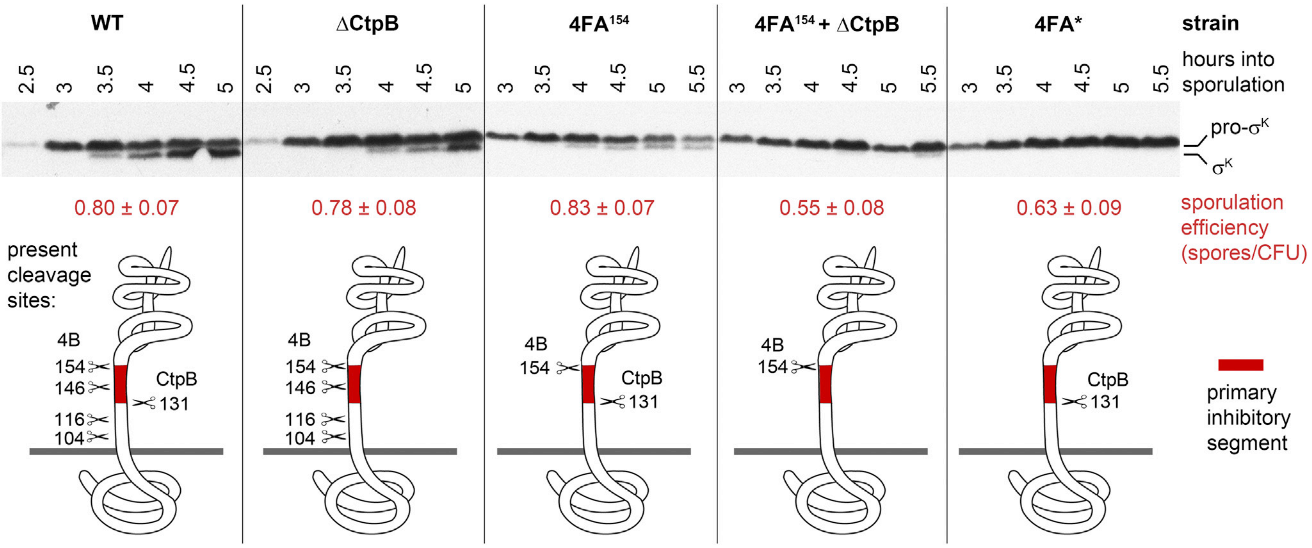

Figure 6. Removal of the 4FA Segment 131-154 by 4B and CtpB Is Critical for Relieving Inhibition of 4FB

After inducing sporulation, *B. subtilis* cell extracts (genotypes indicated) were prepared at defined time points and analyzed by immunoblots using anti-σ^K antibodies. The respective bands for pro-σ^K and σ^K are labeled and sporulation efficiencies are indicated together with the standard errors of the mean (CFU, colony forming unit). The cartoon below illustrates possible cleavage events. According to the in vivo data, the 4FA region 131-154 (highlighted in red), which is removed by the concerted activity of the 4B and CtpB proteases, is the primary inhibitor motif of the I-CLiP 4FB. See also Table S3.

addition, indicate that the removal of the 4FA segment 131-154, either by repeated 4B cleavage or by the concerted 4B-CtpB activities, relieves inhibition of the 4FB RIP protease triggering pro-σ^K processing and consequently spore formation.

DISCUSSION

CtpB Reveals the Mechanistic Versatility of PDZ-Proteases in Signaling Pathways

In this work we determined the crystal structures of CtpB in different functional states, including one resting and three active conformations. A structure-guided mutational analysis led us to propose a model for how the CtpB protease is activated. The two most striking features of this mechanism comprise a PDZ-gated protease tunnel and the reversible switch between resting and activated protease states in response to peptide binding to the PDZ domain. Phenotypically, this allosteric mechanism is similar to the activation of HtrA proteases (Clausen et al., 2011). Though structurally unrelated, both CtpB and HtrA evolved a functional motif (CtpB: protease cap; HtrA: activation domain) that reorients and completes the active site in response to peptide binding to the PDZ domain (Figure S6A). In the case of HtrA proteins, a particular protease loop (L3) functions as a critical switch element that interacts with the PDZ domain and stabilizes the active protease conformation in the presence of substrate (DegP; Kim et al., 2011; Krojer et al., 2008a; Krojer et al., 2008b) or allosteric activator (DegS; Hasselblatt et al., 2007; Sohn and Sauer, 2009; Wilken et al., 2004). In CtpB, the activation signal is communicated to the active site by assigning a dual function to the PDZ residue Arg168, which is critical for binding the C terminus of substrate as well as for stabilizing the active protease. Owing to the close collaboration of protease and PDZ domains in setting up the composite CtpB degradation system, the predigested $4FA_{proc}$ can function as substrate as well as allosteric activator. By binding to the PDZ domain of CtpB, $4FA_{proc}$ opens the protease tunnel thus inducing its own degradation. This *cis*-activation mechanism is different from the regulation of other PDZ-proteases that are typically activated in *trans*, as for example stress-signaling peptides activate DegS to cleave the RseA substrate (Walsh et al., 2003). Moreover, the PDZ domain of CtpB controls protease function in a unique way. Whereas activation of HtrA proteases is under control of a disorder-to-order transition, the PDZ domain of the resting CtpB disrupts the proteolytic site in a structurally defined manner blocking the protease tunnel and separating the catalytic residues. Accordingly, the PDZ domain of CtpB appears to function as an enzymatic prodomain that needs to be reoriented before substrates can be cleaved. Coupling the reorientation of the PDZ prodomain with substrate binding ensures that the CtpB signaling protease can be switched on and off depending on the availability of substrate. Moreover, the PDZ-gated protease tunnel enables CtpB to trim the 4FA substrate in a highly specific manner despite the fact that the targeted protein is largely unstructured. Upon recognizing the C terminus of the preprocessed 4FA, the PDZ domain reorients and pulls the downstream cleavage site, which is located 23 residues away, into the protease tunnel where it gets cleaved. Such specific trimming of an unfolded protein is a unique property of the C-terminal processing proteases demanding for sophisticated regulatory measures as seen for CtpB. Importantly, structural comparison with the related D1P protease suggests that the CtpB mechanism may be generally relevant to regulate tail-specific proteases and respective signaling pathways (Figure S6C). Taken together, the present work highlights the convergent

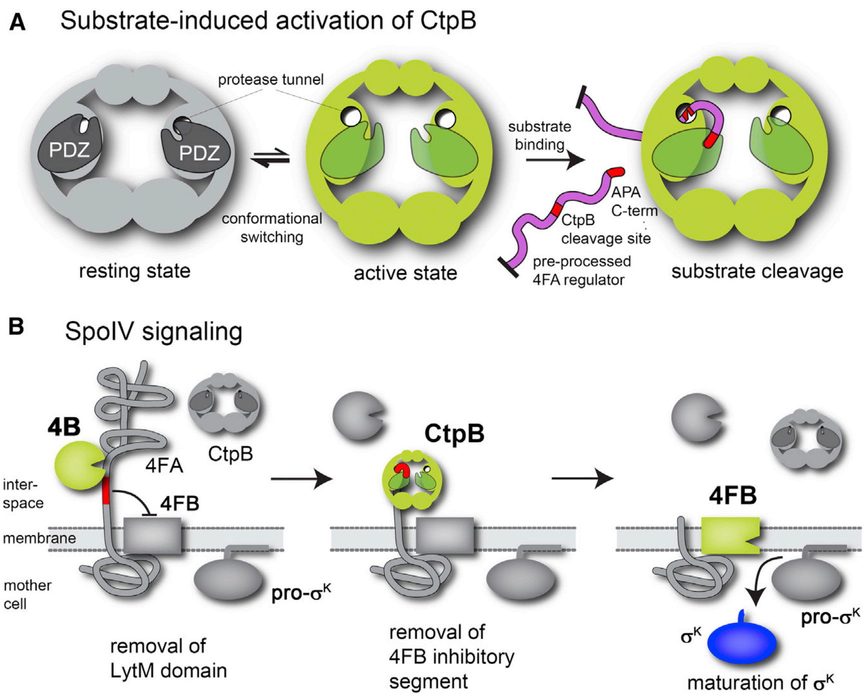

Figure 7. Model for Cooperative Cleavage of 4FA by the Signaling Proteases 4B and CtpB

(A) Cartoon illustrating the allosteric regulation of CtpB. The CtpB protease can reversibly switch between active and resting conformations. When $4FA_{proc}$ (magenta) binds to the PDZ domain, the activated CtpB removes the 4FA linker segment 131-154 protruding through the protease tunnel. CtpB cleavage site (Ala131) and 4B-processed C terminus (Ala152-Pro153-Ala154) are colored red.

(B) The SpoIV proteolytic cascade transmits the sporulation signal from forespore to mother cell. Our data indicate that the three participating proteases 4B, CtpB and 4FB act sequentially in the RIP pathway (respective active forms shown in green). Initially, the 4FA regulator is protected from CtpB cleavage by its folded C-terminal domain. Thus, CtpB activity strictly depends on the previous shedding of 4FA by the 4B protease. The truncated 4FA C terminus binds and activates CtpB, which in turn removes the inhibitory 4FA segment indicated in red. The unreleased I-CLiP membrane protease 4FB can now cleave the membrane-associated pro-σ^K sigma factor, releasing σ^K into the cytosol and allowing sporulation to proceed. See also Figure S6.

evolution of PDZ-proteases to become activated in a reversible but tightly controlled manner by employing PDZ domains as regulatory modules. In this context, the versatile, and strikingly unpredictable, mechanistic role of a PDZ domain when linked to a "simple" protease is remarkable. As seen in great detail for CtpB, this multifaceted function includes substrate sensing, active site gatekeeping and conformational switching. Presumably, it is this functional versatility that underlies the prominent role of PDZ-proteases in controlling diverse cell signaling pathways.

CtpB Assembles the Minimal Version of a Self-Compartmentalizing Protease

The protease fold of CtpB is directly related to the cage-forming ClpP and tricorn proteases, which function as molecular shredders in bacterial protein quality control. For both cage-forming proteins, the proteolytic sites are buried within a self-compartmentalizing particle, in which substrate filtering is achieved by sophisticated gating mechanisms, i.e., a regulated entrance pore in case of ClpP (Gottesman et al., 1997; Lee et al., 2010) and a substrate-guiding β-propeller in the tricorn protease (Brandstetter et al., 2001; Goettig et al., 2005). Our data suggest that CtpB represents a minimal version of a self-compartmentalizing protease that features a narrow tunnel with a built-in substrate filter and a gatekeeping PDZ domain. To identify structural motifs characterizing these distinctly safeguarded proteases, we aligned the active forms of CtpB and ClpP. Even though the two proteases are only distantly related, ClpP and CtpB exhibit conserved mechanistic features including the construction of the oxyanion hole and the substrate specificity pockets (Figure S6C). However, a striking difference relates to the activation of the active site nucleophile. Whereas Ser97 of ClpP is activated by the commonly found Ser-His-Asp catalytic triad, Ser309 of CtpB is part of a Ser-Lys-Gln triad. Importantly, the third residue of the respective triads is contributed by different protease segments. In ClpP, the corresponding aspartate is part of a relatively rigid segment protruding from the core β sheet. In contrast, in CtpB, Gln338 originates from an additional domain, the protease cap that undergoes profound conformational rearrangements during the activation process. Accordingly, the active site architecture of ClpP and CtpB seems to reflect the transformation of a rather static protease cage that is controlled by a remote gating mechanism into a flexible protease tunnel, which is composed of distinct domains and can be allosterically controlled by specific molecular cues (Figure S6B).

Implications for SpoIV RIP Signaling and Cell-Cell Communication Pathways

The present work clarifies the collaboration of the two signaling proteases CtpB and 4B in the SpoIV sporulation pathway revealing a novel design principle of RIP cascades. Previous studies showed that 4B-deficient strains are incapable of relieving inhibition of the I-CLiP protease 4FB and are blocked in the production of mature σ^K required to complete spore formation (Hoa et al., 2002). As these strains express normal levels of the second signaling protease CtpB, it was unclear why CtpB was unable to cleave 4FA and activate pro-σ^K processing. Our data demonstrate that 4B and CtpB closely collaborate in degrading the 4FA inhibitor (Figure 7). The electron density of the copurified ligand bound to the PDZ domain of CtpB is compatible with a Pro-Ala C-terminal motif. Intriguingly, a corresponding C terminus is generated by the 4B protease when cleaving 4FA at its primary cleavage site. Though the $4FA_{proc}$ C terminus is not the preferred ligand of CtpB in vitro, protease and

cocrystallization assays indicate that the weak affinity of the degradation tag is sufficient to activate CtpB. According to these data, the predigested $4FA_{proc}$ substrate may need to reach a certain level to turn on CtpB activity. Such a mechanism would prevent the premature induction of the sporulation pathway and in parallel preclude the unspecific cleavage of nonnative substrates carrying a similar degradation tag. In conclusion, we propose that 4B and CtpB act sequentially on 4FA with the 4B protease first removing the compact LytM domain and then, and only then, CtpB further cleaves 4FA removing the remaining linker region (residues 131-154). According to our in vivo data, it is this linker segment that primarily inhibits the I-CLiP protease 4FB and must be removed to continue the SpoIV RIP pathway.

Why are two signaling proteases involved in degrading the SpoIV regulator protein 4FA? Typically, RIP pathways are linear cascades, in which a site-1 cleavage event sheds an inhibitory ectodomain from the signaling protein and enables the subsequent site-2 cleavage of the very same protein, producing one signaling molecule per cycle (Brown et al., 2000; Wolfe and Kopan, 2004). In sharp contrast, the RIP cascade initiated by 4B and CtpB leads to a constitutively active membrane protease 4FB, which can target several pro-σ^k molecules and thus functions as a signal amplifier in the SpoIV pathway. The pronounced signaling capacity of the 4FB I-CLiP may thus necessitate a more elaborate control in its activation mechanism. As revealed in this work, this mechanism relies on the concerted activity of two intermembrane proteases that ensure the precise temporal control of transducing an activating signal from forespore to mother cell. In addition, the present SpoIV RIP mechanism illustrates how different activation signals can be integrated into one pathway. Dependent on the origin of the signaling proteases such a mechanism could coordinate either different transcriptional programs within a single cell or, in case where the signaling proteases are secreted from two neighboring cells, link the RIP signal cascade to cell-cell communication coordinating for example developmental programs.

EXPERIMENTAL PROCEDURES

Protein Expression and Purification

Recombinant His-tagged proteins were expressed in *E. coli* BL21(DE3)pLysS or B834(DE3) (SeMet derivatized proteins), respectively. All proteins were purified by Ni-affinity chromatography and size exclusion chromatography (SEC) using standard protocols. As final SEC buffer, we used 20 mM Tris (pH 7.5), 150 mM NaCl, 5 mM β-mercapto-ethanol. In vitro transcription, translation reactions were performed using the promega TNT Quick Coupled Transcription/Translation System, following manufactures instructions.

Crystallization and Structure Solution of Resting and Activated CtpB

All CtpB crystals were grown at 19°C using the sitting-drop vapor-diffusion method. Collection of diffraction data, structure solution, and refinement proceeded by standard procedures, as detailed in Supplemental Information.

In Vitro and In Vivo Activity Assays

To follow the protease activity of 4B and CtpB in vitro, the recombinant proteases were incubated with different forms of the 4FA substrate or a *B. subtilis* membrane fraction containing GFP-4FA (Campo and Rudner, 2006) for various times at 25°C. To generate the preprocessed 4FA variant, SUMO-$4FA_{EC}$ was treated for 90 min with 4B. Subsequently, CtpB was added and the reaction prolonged for various times. Reactions were stopped by adding SDS loading buffer, cleavage products separated by SDS–PAGE and visualized by Coomassie staining, ^{35}S-autoradiography or western blotting using anti-GFP antibodies. For in vivo assays, activity of the I-CLiP protease 4FB was monitored by following production of the mature σ^K. In parallel, the efficiency of spore formation was determined upon nutrient exhaustion, as described in (Campo and Rudner, 2006). Plasmids, primers, and strains used in this study are summarized in Tables S4, S5, and S6, respectively.

Peptide Spot Assay

Peptide spot libraries were generated by synthesizing the 11 C-terminal residues of 1,849 *B. subtilis* proteins predicted to reside in the intermembrane space onto a cellulose membrane. Membranes were blocked for 4 hr with blocking buffer (blocking reagent, Sigma-Genosys) and subsequently incubated with CtpB. Bound protein was identified using specific antibodies. Further libraries were generated, where identified peptide sequences were systematically permutated.

Isothermal Titration Calorimetry

Thermodynamic values characterizing the interaction between CtpB and peptide ligands were determined by ITC (MCS-ITC; Microcal). All experiments were performed in overflow mode at 30°C applying standard procedures with proteins and peptide ligands equilibrated in SEC buffer.

ACCESSION NUMBERS

The Protein Data Bank accession numbers for the coordinates and structure factors reported in this paper are CtpBa1, 4c2c; CtpBa2/3, 4c2d; CtpBi, 4c2e; R168A, 4c2f; S309A/VPA, 4c2g; and V118Y, 4c2h.

SUPPLEMENTAL INFORMATION

Supplemental Information includes Extended Experimental Procedures, six figures, and six tables and can be found with this article online at http://dx.doi.org/10.1016/j.cell.2013.09.050.

AUTHOR CONTRIBUTIONS

M.M., A.H., R.K., and T.C. performed the structural studies, M.M., A.H., R.K. and M.E. performed the protein biochemistry, A.H., P.B., and R.V. carried out the peptide spot analysis, and M.M., C.R., and D.Z.R. the *B. subtilis* experiments. T.C. outlined the work and wrote the manuscript with contribution from all authors.

ACKNOWLEDGMENTS

We are grateful to the beamline staff at the DESY, SLS, and ESRF for their assistance during data collection and Mathias Madalinski for peptide synthesis. We thank members of the Clausen lab for support and encouragement. This work was supported by grants from the Austrian Science Fund (W1221 "DK Structure and Interaction of Biological Macromolecules" to M.M.), EMBO Long-Term Fellowship (A.H.), Deutsche Forschungsgemeinschaft (M.E.; VO885/2 to R.V and P.B.), and the National Institute of Health (GM086466 to D.Z.R.). The IMP is funded by Boehringer Ingelheim.

Received: March 27, 2013
Revised: July 18, 2013
Accepted: September 18, 2013
Published: October 24, 2013

REFERENCES

Boisguerin, P., Ay, B., Radziwill, G., Fritz, R.D., Moelling, K., and Volkmer, R. (2007). Characterization of a putative phosphorylation switch: adaptation of SPOT synthesis to analyze PDZ domain regulation mechanisms. ChemBioChem *8*, 2302–2307.

Brandstetter, H., Kim, J.S., Groll, M., and Huber, R. (2001). Crystal structure of the tricorn protease reveals a protein disassembly line. Nature *414*, 466–470.

Brown, M.S., Ye, J., Rawson, R.B., and Goldstein, J.L. (2000). Regulated intramembrane proteolysis: a control mechanism conserved from bacteria to humans. Cell *100*, 391–398.

Campo, N., and Rudner, D.Z. (2006). A branched pathway governing the activation of a developmental transcription factor by regulated intramembrane proteolysis. Mol. Cell *23*, 25–35.

Campo, N., and Rudner, D.Z. (2007). SpoIVB and CtpB are both forespore signals in the activation of the sporulation transcription factor sigmaK in Bacillus subtilis. J. Bacteriol. *189*, 6021–6027.

Clausen, T., Kaiser, M., Huber, R., and Ehrmann, M. (2011). HTRA proteases: regulated proteolysis in protein quality control. Nat. Rev. Mol. Cell Biol. *12*, 152–162.

Cutting, S., Oke, V., Driks, A., Losick, R., Lu, S., and Kroos, L. (1990). A forespore checkpoint for mother cell gene expression during development in B. subtilis. Cell *62*, 239–250.

Errington, J. (2003). Regulation of endospore formation in Bacillus subtilis. Nat. Rev. Microbiol. *1*, 117–126.

Fokine, A., Miroshnikov, K.A., Shneider, M.M., Mesyanzhinov, V.V., and Rossmann, M.G. (2008). Structure of the bacteriophage phi KZ lytic transglycosylase gp144. J. Biol. Chem. *283*, 7242–7250.

Goettig, P., Brandstetter, H., Groll, M., Göhring, W., Konarev, P.V., Svergun, D.I., Huber, R., and Kim, J.S. (2005). X-ray snapshots of peptide processing in mutants of tricorn-interacting factor F1 from Thermoplasma acidophilum. J. Biol. Chem. *280*, 33387–33396.

Gottesman, S., Maurizi, M.R., and Wickner, S. (1997). Regulatory subunits of energy-dependent proteases. Cell *91*, 435–438.

Hara, H., Yamamoto, Y., Higashitani, A., Suzuki, H., and Nishimura, Y. (1991). Cloning, mapping, and characterization of the Escherichia coli prc gene, which is involved in C-terminal processing of penicillin-binding protein 3. J. Bacteriol. *173*, 4799–4813.

Hasselblatt, H., Kurzbauer, R., Wilken, C., Krojer, T., Sawa, J., Kurt, J., Kirk, R., Hasenbein, S., Ehrmann, M., and Clausen, T. (2007). Regulation of the sigmaE stress response by DegS: how the PDZ domain keeps the protease inactive in the resting state and allows integration of different OMP-derived stress signals upon folding stress. Genes Dev. *21*, 2659–2670.

Hoa, N.T., Brannigan, J.A., and Cutting, S.M. (2002). The Bacillus subtilis signaling protein SpoIVB defines a new family of serine peptidases. J. Bacteriol. *184*, 191–199.

Keiler, K.C., Waller, P.R., and Sauer, R.T. (1996). Role of a peptide tagging system in degradation of proteins synthesized from damaged messenger RNA. Science *271*, 990–993.

Kim, S., Grant, R.A., and Sauer, R.T. (2011). Covalent linkage of distinct substrate degrons controls assembly and disassembly of DegP proteolytic cages. Cell *145*, 67–78.

Krojer, T., Pangerl, K., Kurt, J., Sawa, J., Stingl, C., Mechtler, K., Huber, R., Ehrmann, M., and Clausen, T. (2008a). Interplay of PDZ and protease domain of DegP ensures efficient elimination of misfolded proteins. Proc. Natl. Acad. Sci. USA *105*, 7702–7707.

Krojer, T., Sawa, J., Schäfer, E., Saibil, H.R., Ehrmann, M., and Clausen, T. (2008b). Structural basis for the regulated protease and chaperone function of DegP. Nature *453*, 885–890.

Kroos, L., Kunkel, B., and Losick, R. (1989). Switch protein alters specificity of RNA polymerase containing a compartment-specific sigma factor. Science *243*, 526–529.

Lee, B.G., Park, E.Y., Lee, K.E., Jeon, H., Sung, K.H., Paulsen, H., Rübsamen-Schaeff, H., Brötz-Oesterhelt, H., and Song, H.K. (2010). Structures of ClpP in complex with acyldepsipeptide antibiotics reveal its activation mechanism. Nat. Struct. Mol. Biol. *17*, 471–478.

Liao, D.I., Qian, J., Chisholm, D.A., Jordan, D.B., and Diner, B.A. (2000). Crystal structures of the photosystem II D1 C-terminal processing protease. Nat. Struct. Biol. *7*, 749–753.

Losick, R., and Stragier, P. (1992). Crisscross regulation of cell-type-specific gene expression during development in B. subtilis. Nature *355*, 601–604.

Morgunova, E., Tuuttila, A., Bergmann, U., Isupov, M., Lindqvist, Y., Schneider, G., and Tryggvason, K. (1999). Structure of human pro-matrix metalloproteinase-2: activation mechanism revealed. Science *284*, 1667–1670.

Pan, Q., Losick, R., and Rudner, D.Z. (2003). A second PDZ-containing serine protease contributes to activation of the sporulation transcription factor sigmaK in Bacillus subtilis. J. Bacteriol. *185*, 6051–6056.

Rawlings, N.D., O'Brien, E., and Barrett, A.J. (2002). MEROPS: the protease database. Nucleic Acids Res. *30*, 343–346.

Resnekov, O., and Losick, R. (1998). Negative regulation of the proteolytic activation of a developmental transcription factor in Bacillus subtilis. Proc. Natl. Acad. Sci. USA *95*, 3162–3167.

Rudner, D.Z., and Losick, R. (2002). A sporulation membrane protein tethers the pro-sigmaK processing enzyme to its inhibitor and dictates its subcellular localization. Genes Dev. *16*, 1007–1018.

Rudner, D.Z., Fawcett, P., and Losick, R. (1999). A family of membrane-embedded metalloproteases involved in regulated proteolysis of membrane-associated transcription factors. Proc. Natl. Acad. Sci. USA *96*, 14765–14770.

Setlow, P. (1995). Mechanisms for the prevention of damage to DNA in spores of Bacillus species. Annu. Rev. Microbiol. *49*, 29–54.

Sohn, J., and Sauer, R.T. (2009). OMP peptides modulate the activity of DegS protease by differential binding to active and inactive conformations. Mol. Cell *33*, 64–74.

Stragier, P., and Losick, R. (1996). Molecular genetics of sporulation in Bacillus subtilis. Annu. Rev. Genet. *30*, 297–41.

Takahashi, M., Shiraishi, T., and Asada, K. (1988). COOH-terminal residues of D1 and the 44 kDa CPa-2 at spinach photosystem II core complex. FEBS Lett. *240*, 6–8.

Walsh, N.P., Alba, B.M., Bose, B., Gross, C.A., and Sauer, R.T. (2003). OMP peptide signals initiate the envelope-stress response by activating DegS protease via relief of inhibition mediated by its PDZ domain. Cell *113*, 61–71.

Wilken, C., Kitzing, K., Kurzbauer, R., Ehrmann, M., and Clausen, T. (2004). Crystal structure of the DegS stress sensor: How a PDZ domain recognizes misfolded protein and activates a protease. Cell *117*, 483–494.

Wolfe, M.S., and Kopan, R. (2004). Intramembrane proteolysis: theme and variations. Science *305*, 1119–1123.

Zhou, R., and Kroos, L. (2005). Serine proteases from two cell types target different components of a complex that governs regulated intramembrane proteolysis of pro-sigmaK during Bacillus subtilis development. Mol. Microbiol. *58*, 835–846.

Supplemental Information

EXTENDED EXPERIMENTAL PROCEDURES

Protein Expression and Purification

For protein expression, BL21(DE3)pLysS cells were transformed with the respective plasmids and grown in LB medium at 37°C to an OD_{600} of ~0.8. Expression was induced by the addition of 0.5 mM IPTG and cells were harvested after 90 min. To produce selenomethionine modified CtpB, B834(DE3) cells that are impaired in methionine biosynthesis were grown in a corresponding MinA medium. To isolate His-tagged proteins, the cells were harvested by centrifugation and pellets dissolved in buffer A (CtpB and 4FA: 20 mM Tris-HCl [pH 7.5], 150 mM NaCl, 5 mM β-mercapto-ethanol; 4B: 20 mM Na_2HPO_4 [pH 7.5], 300 mM NaCl). Subsequently, cells were broken by sonication and the cleared lysate was applied to a HisTrap Ni-affinity column and proteins eluted with a stepwise imidazol gradient. CtpB and 4FA variants were further purified by size exclusion chromatography (SEC) using a 26/60 S200 or S75 column, respectively, equilibrated with buffer B (10 mM Tris [pH 7.5], 150 mM NaCl, 5 mM β-mercaptoethanol). For 4B, the buffer of the Ni-NTA eluate was adjusted to 20 mM Tris-HCl [pH 9], 50 mM NaCl using a 26/10 G-25 desalting column prior to purifying the sample by Resource-Q anion exchange chromatography. The 4B protease was collected from the flow-through, concentrated and applied to a SEC column equilibrated with buffer B.

In Vitro Transcription, Translation, and ^{35}S Labeling

$4FA_{proc}$ with alternative C-termini was produced using the promega TNT Quick Coupled Transcription/Translation System, following manufactures instructions. Briefly, 40 μl of TNT Master Mix were added to 0.5 μg of template plasmid DNA and 20 μCi of ^{35}S-methionine (Perkinelmer). The coupled transcription-translation reaction was carried out upon incubation at 30°C for 2 hr. Relative product amounts were normalized based on ^{35}S-autoradiography following SDS-PAGE analysis.

Crystallization and Data Collection

CtpB crystals were grown at 19°C by the sitting-drop vapor-diffusion method using a 2:1 mixture of protein (20 mg/ml) and reservoir solution. To obtain S309A/VPA crystals, CtpB (20 mg/ml) was incubated with 1 mM peptide (NH_2-EMDKPQTAAVPA-COOH) for 1 hr at 19°C before setting up crystal trials. Crystals were grown using the following reservoir solutions: (CtpBa1, R168A and S309A/VPA: 2.1 M Na-malonate [pH 7.8]; CtpBa2/3: 13% PEG 3350, 0.1 M Na-malonate, 0.1 M HEPES [pH 7.8]; CtpBi: 10% isopropanol, 0.1 M imidazol [pH 8.0]; V118Y: 15% 2-methyl-2,4-pentanediol, 5% PEG 4000, 0.1 M Imidazol [pH 8.0]). For data collection crystals were incubated in cryoprotectant solution and flash-frozen in liquid nitrogen (CtpBa1, R168A and CtpBi/VPA: cryoprotectant was identical to reservoir solution; CtpBa2/3, CtpBi and V118Y: for cryoprotection, the reservoir solution was supplemented with 20% 2-methyl-2,4-pentanediol). High-resolution diffraction data of CtpBa1 and R168A were collected at the Swiss Light Source (SLS, beamline X06DA) at λ = 0.9791 Å using a Pilatus detector (Dectris), whereas diffraction data of CtpBa2/3, V118Y and CtpBi were collected at the European Synchrotron Radiation Facility (ESRF, Grenoble, France) beamline ID 14-4. Finally, diffraction data of CtpBi/VPA was collected at Deutsches Elektronen-Synchrotron (DESY, beamline P14) using a Pilatus detector (Dectris). Integration and scaling of diffraction data were performed with the programs DENZO, and SCALEPACK (Minor, 1997). The crystal parameters are summarized in Table S1.

Structure Determination and Refinement

For CtpBa1, 10 out of 11 selenium sites were localized with SHELXD (Sheldrick, 2008) and refined with SHARP (Bricogne et al., 2003). Solvent flattening was performed with the program SOLOMON (Abrahams and Leslie, 1996). For all other structures, phases were determined by molecular replacement using the program PHASER (McCoy et al., 2007) and the structure of CtpBa1 as search model. In all cases, energy restrained refinement was performed with the programs CNS (Brünger et al., 1998) and PHENIX (Adams et al., 2010), utilizing bulk solvent and anisotropic B-factor corrections depending on the development of the R_{free} index. Refinement was performed in multiple cycles, interrupted for manual model re-building with the program O (Jones et al., 1991). Final models were verified with the program MolProbity (Chen et al., 2010) and crystal contacts analyzed using the PISA algorithm implemented on the Protein Data Bank Europe website (Krissinel and Henrick, 2007). Molecular illustrations were prepared with PYMOL (Schrodinger, 2010).

Sequence and Structure Alignments

Sequence alignments of CtpB proteases were prepared with the program ClustalW (Chenna et al., 2003) and visualized using the ESPript server (Gouet et al., 1999). Structural alignments analyzing the protease core of CtpB were performed with the structure based algorithm cealign implemented in PYMOL (Schrodinger, 2010). Root-mean-square deviations were calculated by pair-wise fitting of equivalent Cα atoms. The residue numbers of equivalent Cα's are as follows: D1P 278-333, 334-355 and 357-405 fitted to CtpB 215-270, 272-293 and 294-342; ClpP 27-32, 34-54, 57-63, 65-79, 83-106, 110-114 and 117-129 fitted to CtpB 215-220, 221-241, 243-249, 252-266, 295-318, 325-329 and 330-342; tricorn 880-903, 904-919, 920-929 and 951-998 fitted to CtpB 215-238, 241-256, 259-268 and 295-342.

Preparation of Solubilized GFP-4FA from *B. subtilis* Membranes

Extraction of *B. subtilis* membrane proteins was performed as described previously (Campo and Rudner, 2006). In brief, *B. subtilis* cultures (strain BNC694), expressing a GFP-4FA fusion protein, were grown to an OD_{600} of 0.6 in CH medium. Sporulation was

induced in resuspension media and cells harvested after 4 hr by centrifugation. Resulting pellets were washed twice with SMM buffer (0.5 M Sucrose, 20 mM $MgCl_2$, 20 mM maleic acid [pH 6.5]) and finally protoplasted by resuspension in SMM buffer supplemented with 0.5 mg/ml lysozyme. Protoplasts were collected by centrifugation and subjected to osmotic lysis in a hypotonic buffer. Lysates were applied to ultracentrifugation at 100,000xg for 1 hr at 4°C. Resulting pellets containing crude membrane preparations were dispersed in hypertonic buffer containing *E. coli* total lipid extract (Avanti) and 0.5% digitonin. The suspension was incubated for 1 hr at 4°C to extract membrane proteins. Tables S4, S5, and S6 summarize the used plasmids and strains.

In Vitro Cleavage Assays

To measure the proteolytic activities of the SpoIV signaling proteases, recombinant, purified proteases (CtpB, 4B or both, 3 μM) were mixed with respective 4FA model substrates (15 μM) and incubated for various times at 25°C. Reactions were stopped by adding SDS loading buffer. Subsequently, samples were incubated for 5 min at 95°C and analyzed by SDS-PAGE and Coomassie staining. To test the activity of CtpB in cleaving the 4B-predigested 4FA substrate, SUMO-$4FA_{EC}$ (15 μM) was incubated with 4B (15 μM) in buffer B for 90 min at 25°C. Subsequently, CtpB (wild-type or mutant forms, 0.3 μM) were added and incubated for various times at 25°C. Reaction aliquots were stopped and analyzed as described. To compare the cleavage efficiency of various CtpB mutants, the amount of $4FA_{proc}$ substrate was quantified by analyzing the stained gels using the software ImageJ (Schneider et al., 2012). To test the cleavage susceptibility of membrane-embedded 4FA, digitonin-solubilized membrane proteins were incubated with the purified 4B and CtpB proteases (3 μM each). Reactions were analyzed by SDS–PAGE and immunoblotting using anti-GFP antibodies (Rudner and Losick, 2002). For cleavage assays testing the degradation of in vitro translated, ^{35}S-labeled $4FA_{proc}$ substrates, 0.3 μM of CtpB (wild-type or ΔPDZ) were directly incubated with the in vitro translation reactions. The cleavage assays were carried out as described and analyzed by SDS-PAGE and ^{35}S-autoradiography using Amercham Hyperfilm™ MP films.

In Vivo Cleavage Assay

As described in (Campo and Rudner, 2006), shedding of the 4FA inhibitor by the 4B and CtpB proteases was characterized in vivo by monitoring pro-σ^K cleavage carried out by the concurrently activated I-CLiP 4FB. In brief, *B. subtilis* strains carrying specific mutations in 4FA and/or CtpB were grown in CH medium to OD_{600} = 0.6 at 37°C. To induce sporulation, cells were harvested by centrifugation at room temperature and resuspended in an equal volume of a low-nutrient resuspension medium. The culture was further incubated at 37°C. At various times, 1 ml fractions were taken, sedimented and the pellets stored at −80°C. For western blot analysis, whole-cell extracts were prepared by dissolving pellets in 50 μl lysis buffer. Upon adding SDS-loading buffer, samples were incubated at 95°C and analyzed by SDS-PAGE and immunoblotting using anti-σ^K antibodies (Resnekov et al., 1996).

Determination of Sporulation Efficiency

Sporulation efficiency of *B. subtilis* strains was determined 24 hr after induction of spore formation by nutrient exhaustion. Sporulated cultures were serially diluted in T-Base medium and subsequently divided into two fractions. To obtain the total number of colony forming units (CFU) within the culture, one fraction was directly plated on DSM-agar and incubated over night at 37°C. To determine the number of spores within the culture, the second fraction was subjected to a 20 min heat-treatment at 85°C, killing all nonsporulated cells, before also being plated on DSM-agar and incubated over night at 37°C. Colonies formed by both fractions were counted, and sporulation efficiency calculated as number of spores per CFU. Results are summarized in Table S3.

Peptide Spot Assay

Peptide spot libraries were generated by synthesizing the 11 C-terminal residues of 1849 *B. subtilis* proteins known to reside in the intermembrane space. Peptide libraries were generated by a MultiPep SPOTrobot (INTAVIS Bioanalytical Instruments AG) using an optimized method for generating inverted peptides (Boisguerin et al., 2007; Volkmer, 2009). The peptide array was prewashed with EtOH (1x10 min), washed with Tris-buffered saline (TBS, pH 8.0, 3x10 min), and blocked for 4 hr with blocking buffer (blocking reagent, Sigma-Genosys) in TBS (pH 8.0) containing 5% sucrose. Membranes were incubated with CtpB variants (ΔPDZ or isolated PDZ domain, 10 μg/ml) overnight at 4°C, and then washed with TBS (pH 8.0, 3x10 min). Bound CtpB was detected using a mouse anti-polyHis antibody (Sigma, 1:3,000 in blocking buffer, 2 hr at RT) followed by horseradish-peroxidase conjugated anti-mouse antibody (Calbiochem, 1:2,000 in blocking buffer, 1 hr at RT). Detection was carried out with Uptilight HRP blot chemiluminescent substrate (Uptima) with an exposure time of 5-10 min. The signal intensities with background-subtraction were recorded as biochemical light units with a LumiImager (Boehringer) and analyzed using the LumiAnalyst software. Secondary libraries for substitutional analyses were generated by systematically permutating identified peptides and analyzed by the described peptide spot methodology.

Isothermal Titration Calorimetry

The interaction between CtpB (wild-type; mutant forms or isolated PDZ domain) and peptide ligands was characterized by ITC measurements (MCS-ITC; Microcal). Peptides were dissolved to a concentration of 300 μM in buffer B (10 mM Tris [pH 7.5], 150 mM NaCl, 5 mM β-Mercaptoethanol) and titrated to a 30 μM solution of CtpB present in the same buffer. For all experiments, the injection volume was set to 2 μl for the first and 10 μl for all subsequent injections, the spacing time between the injections was 120 s and the stirring speed 300 rpm. Control experiments were carried out to correct for dilution effects upon buffer titration. All experiments

were performed in overflow mode at 30°C. Resulting data were analyzed with the MicroCal ORIGIN software, following the instructions of the manufacturer. All peptides were synthesized in-house on a Syro Peptide Synthesizer (Multisyntech) and purified by HPLC.

SUPPLEMENTAL REFERENCES

Abrahams, J.P., and Leslie, A.G. (1996). Methods used in the structure determination of bovine mitochondrial F1 ATPase. Acta Crystallogr. D Biol. Crystallogr. *52*, 30–42.

Adams, P.D., Afonine, P.V., Bunkóczi, G., Chen, V.B., Davis, I.W., Echols, N., Headd, J.J., Hung, L.W., Kapral, G.J., Grosse-Kunstleve, R.W., et al. (2010). PHENIX: a comprehensive Python-based system for macromolecular structure solution. Acta Crystallogr. D Biol. Crystallogr. *66*, 213–221.

Bricogne, G., Vonrhein, C., Flensburg, C., Schiltz, M., and Paciorek, W. (2003). Generation, representation and flow of phase information in structure determination: recent developments in and around SHARP 2.0. Acta Crystallogr. D Biol. Crystallogr. *59*, 2023–2030.

Brünger, A.T., Adams, P.D., Clore, G.M., DeLano, W.L., Gros, P., Grosse-Kunstleve, R.W., Jiang, J.S., Kuszewski, J., Nilges, M., Pannu, N.S., et al. (1998). Crystallography & NMR system: A new software suite for macromolecular structure determination. Acta Crystallogr. D Biol. Crystallogr. *54*, 905–921.

Chen, V.B., Arendall, W.B., 3rd, Headd, J.J., Keedy, D.A., Immormino, R.M., Kapral, G.J., Murray, L.W., Richardson, J.S., and Richardson, D.C. (2010). MolProbity: all-atom structure validation for macromolecular crystallography. Acta Crystallogr. D Biol. Crystallogr. *66*, 12–21.

Chenna, R., Sugawara, H., Koike, T., Lopez, R., Gibson, T.J., Higgins, D.G., and Thompson, J.D. (2003). Multiple sequence alignment with the Clustal series of programs. Nucleic Acids Res. *31*, 3497–3500.

Gouet, P., Courcelle, E., Stuart, D.I., and Métoz, F. (1999). ESPript: analysis of multiple sequence alignments in PostScript. Bioinformatics *15*, 305–308.

Jones, T.A., Zou, J.Y., Cowan, S.W., and Kjeldgaard, M. (1991). Improved methods for building protein models in electron density maps and the location of errors in these models. Acta Crystallogr. A *47*, 110–119.

Keiler, K.C., and Sauer, R.T. (1995). Identification of active site residues of the Tsp protease. J. Biol. Chem. *270*, 28864–28868.

Krissinel, E., and Henrick, K. (2007). Inference of macromolecular assemblies from crystalline state. J. Mol. Biol. *372*, 774–797.

McCoy, A.J., Grosse-Kunstleve, R.W., Adams, P.D., Winn, M.D., Storoni, L.C., and Read, R.J. (2007). Phaser crystallographic software. J. Appl. Cryst. *40*, 658–674.

Minor, Z.O.W. (1997). Processing of X-ray Diffraction Data Collected in Oscillation Mode. Methods Enzymol. *276*, 307–326.

Resnekov, O., Alper, S., and Losick, R. (1996). Subcellular localization of proteins governing the proteolytic activation of a developmental transcription factor in *Bacillus subtilis*. Genes Cells *1*, 529–542.

Schneider, C.A., Rasband, W.S., and Eliceiri, K.W. (2012). NIH Image to ImageJ: 25 years of image analysis. Nat. Methods *9*, 671–675.

Schrodinger, L.L.C. (2010). The PyMOL Molecular Graphics System.

Sheldrick, G.M. (2008). A short history of SHELX. Acta Crystallogr. A *64*, 112–122.

Volkmer, R. (2009). Synthesis and application of peptide arrays: quo vadis SPOT technology. ChemBioChem *10*, 1431–1442.

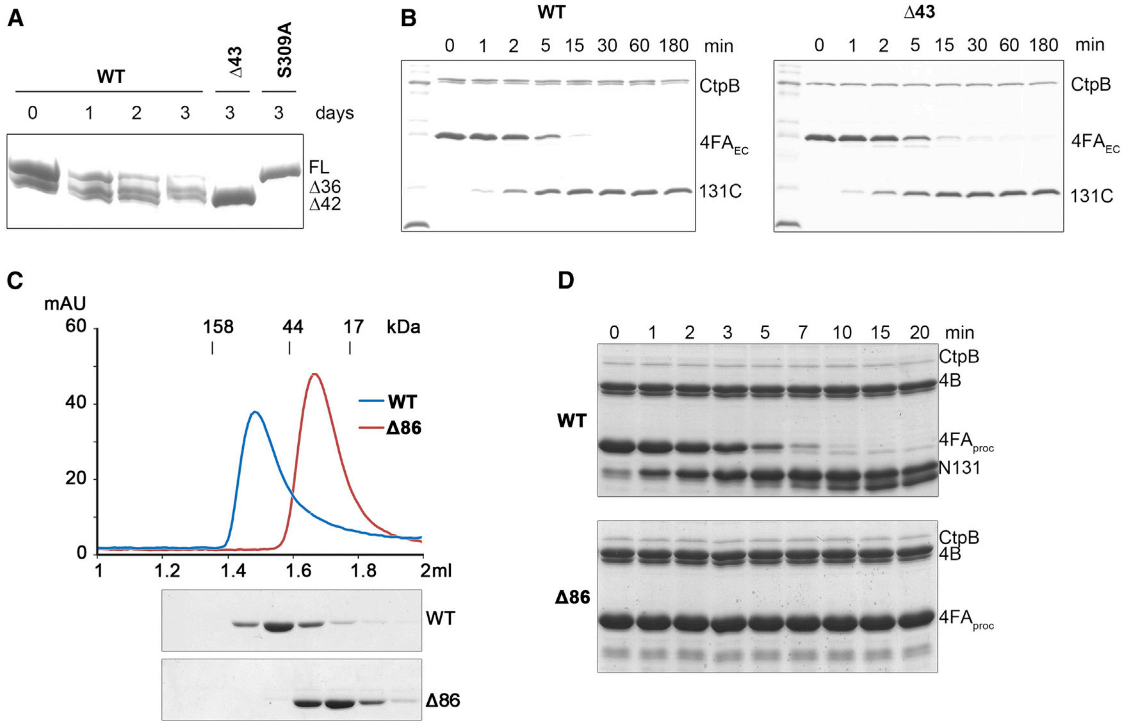

Figure S1. CtpB Autocleavage and Dimerization, Related to Figures 1 and 2

(A) SDS-PAGE analysis monitoring auto-degradation of CtpB. During three days at room-temperature, wild-type CtpB becomes auto-degraded at position 36 and 42, as revealed by mass spectrometry. In contrast, no further degradation is observed in a CtpB construct starting at residue 43 (Δ43) or in the inactive $CtpB_{S309A}$ mutant. (B) Cleavage reactions using the $4FA_{EC}$ substrate show that wild-type and Δ43 CtpB have virtual identical protease activities. (C) Comparison of the gelfiltration profiles of wild-type CtpB and a Δ86 construct lacking the N-terminal dimerization domain. An SDS-PAGE analysis is shown below the chromatograms. (D) Cleavage assays comparing the activity of wild-type and Δ86 CtpB against the 4B predigested $4FA_{proc}$ substrate.

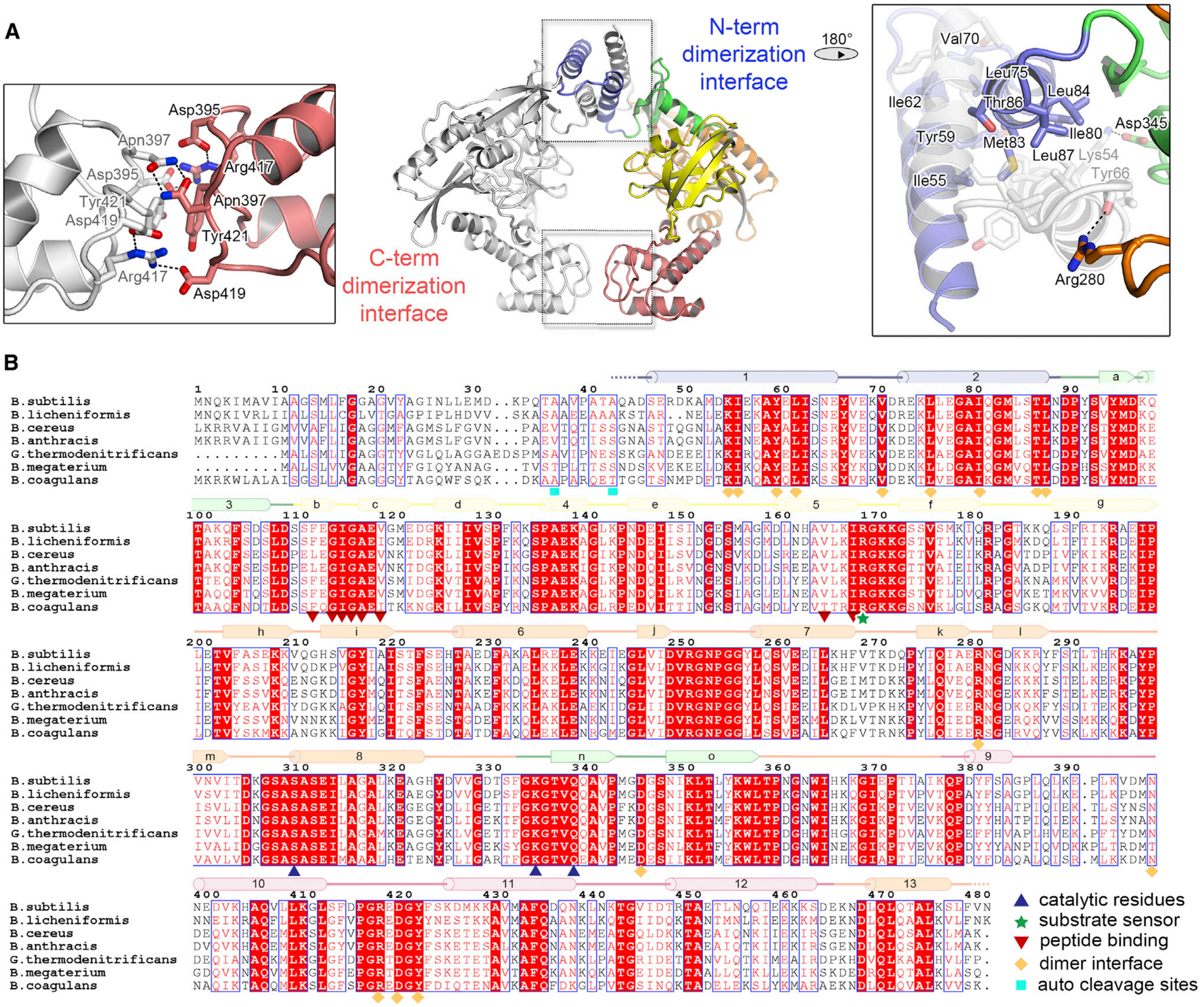

Figure S2. Dimerization Interfaces and Sequence Conservation of CtpB, Related to Figure 2

(A) Detailed view of the dimerization interfaces of CtpB. Residues that mediate direct interactions are labeled and shown in stick representation. The left insert shows the highly polar interface connecting the C-terminal dimerization domains. The right insert illustrates the 4-helix bundle formed by the N-terminal dimerization motifs. (B) Multiple sequence alignment of CtpB from various Gram-positive bacteria. Fully conserved residues are shown with red background; highly conserved residues in red letters. Secondary structure elements are indicated and colored according to the respective domains. Mechanistic important residues are marked with different symbols.

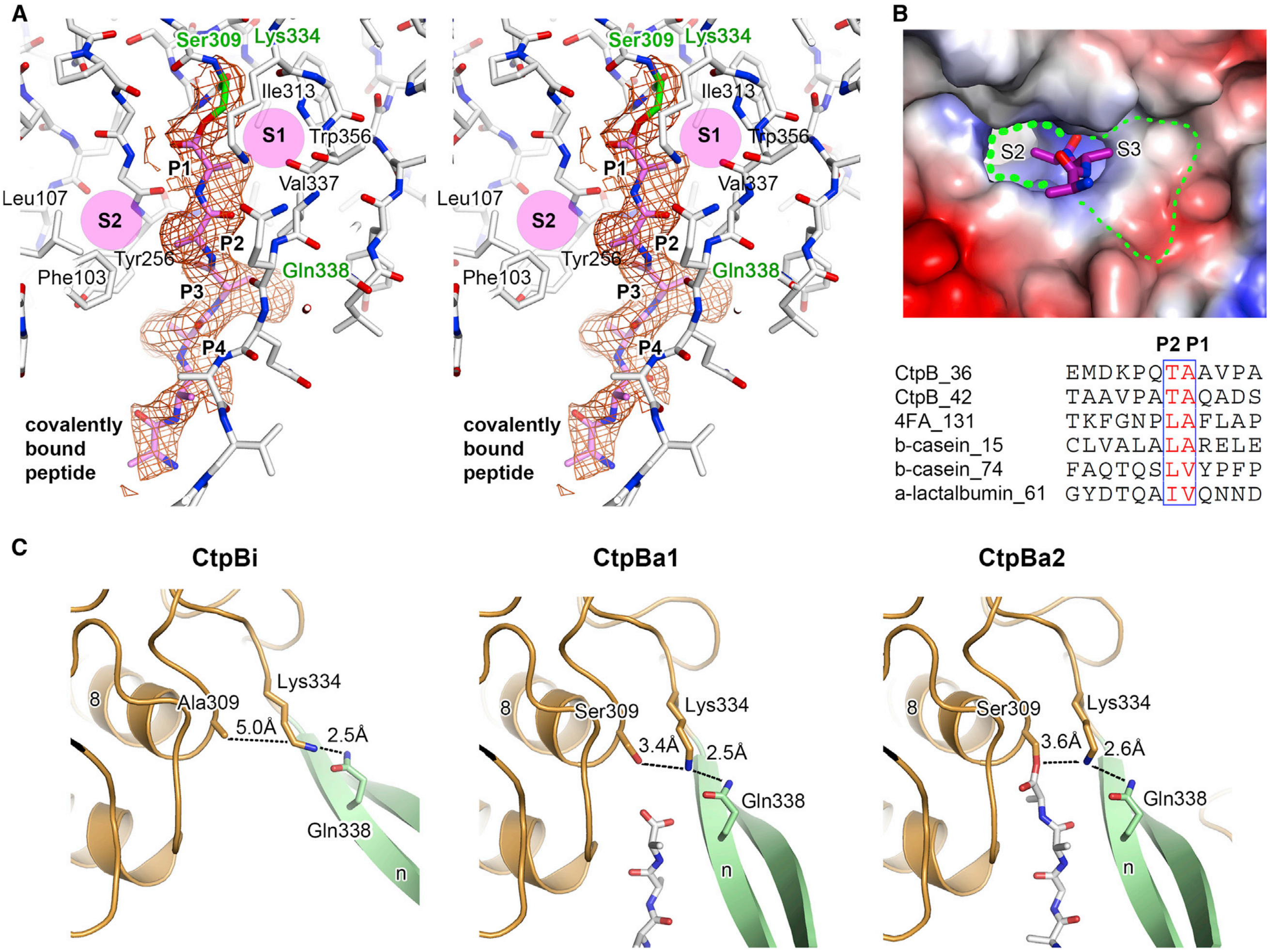

Figure S3. Active site Architecture Revealed By Cocrystallized Peptide Ligands, Related to Figure 3

(A) Stereo view on the active site architecture of CtpBa2 highlighting the peptide ligand (modeled as poly-alanine) that is covalently bound to Ser309. The corresponding 2FoFc omit electron density map is calculated at 2.7 Å and contoured at 1.0 σ. Residues defining the substrate specificity pocket (S1 and S2) are labeled. (B) Molecular surface at the entrance of the protease tunnel with mapped electrostatic potential. The cocrystallized peptide (magenta) defines the specificity pockets of the proteolytic site. In contrast to the S1 and S2 pockets, the S3 pocket appears to be relatively large and unselective. Consistent with this, the identified CtpB cleavage sites shown in the bottom panel have specific P1 and P2 residues, but are otherwise very diverse. (C) Arrangement of the catalytic triad (Ser309, Lys334, Gln338) in distinct functional states. Distances between functional groups are given and the cocrystallized peptides are shown in stick mode.

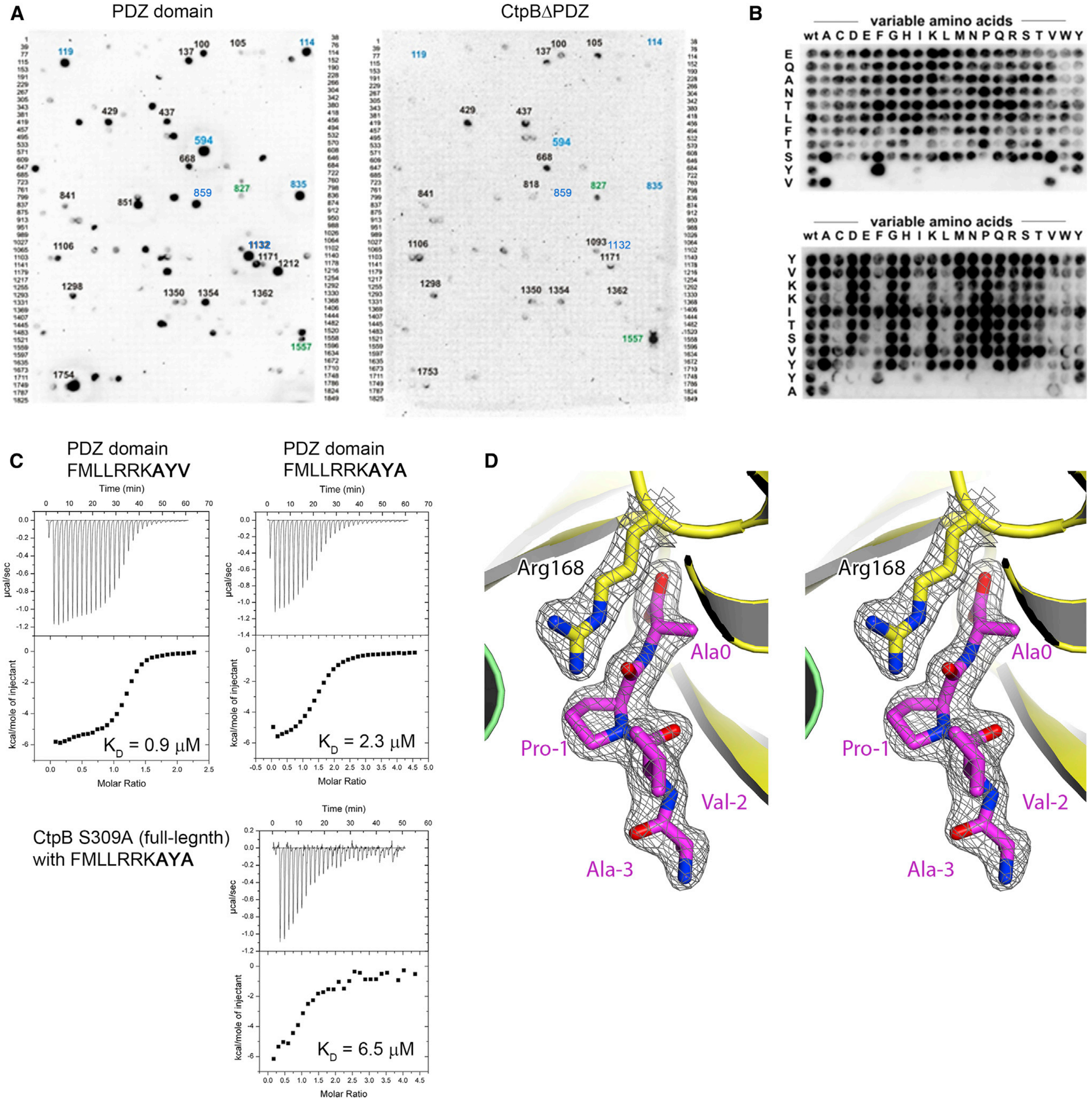

Figure S4. Peptide-Binding Properties of the PDZ Domain, Related to Figure 4

(A) The C termini of *B. subtilis* proteins predicted to reside in the intermembrane space were synthesized on a cellulose membrane according to the method of inverted peptides. Spotted membranes were probed with either the PDZ domain of CtpB (left) or the ΔPDZ mutant (right). (B) Identified strong binding peptides were further analyzed by systematically substituting residues critical for binding to the PDZ domain of CtpB. (C) ITC analysis testing the interaction of the indicated peptides with the isolated PDZ domain and full-length CtpB (S309A). Raw data (top) and binding isotherm derived from the integrated heat (bottom) are shown. The determined K_D dissociation constants are indicated below the binding curves. (D) Stereo view of the C-terminal Val-Pro-Ala-COOH motif that is accommodated in the PDZ domain of CtpB (S309A). Arg168 obtains an important role in tethering the backbone of the penultimate proline residue. The final model is overlaid with the omit 2Fo-Fc electron density (1.9 Å resolution, contoured at 1.2 σ) calculated without peptide ligand and Arg168.

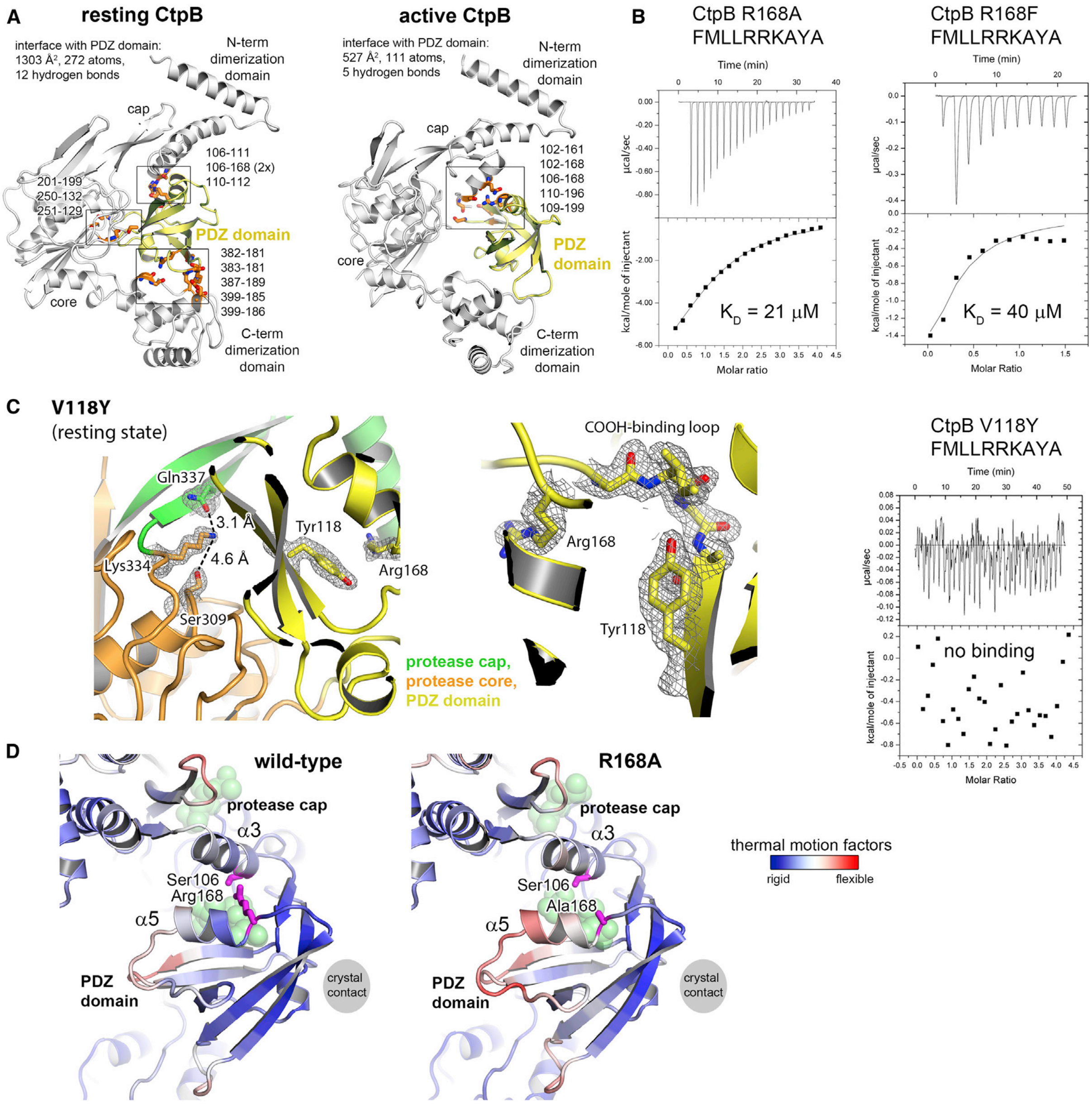

Figure S5. Peptide Binding to the PDZ Domain Stabilizes the Active Protease State of CtpB, Related to Figure 5

(A) The interfaces of the PDZ domain with the other domains of CtpB were analyzed to evaluate the prevailing conformation of CtpB in solution. The ribbon representations of CtpBi (left) and CtpBa1 (right) emphasize the PDZ domain that is shown in yellow. Interactions stabilizing the respective domain interfaces (summarized in top panel) are highlighted in orange and the residues forming hydrogen-bonds are listed. Interactions of the PDZ domain with protease core and C-terminal dimerization domain are only present in the resting state yielding a markedly increased domain interface. Accordingly, the resting state should be the prevailing conformation in solution, as also suggested by the protease assays. (B) ITC analysis characterizing the interaction of a peptide having an Ala-Tyr-Ala C terminus with the full-length CtpB variants R168A, R168F and V118Y. Raw data (top) and binding isotherm derived from the integrated heat (bottom) are shown. The determined K_D dissociation constants are indicated below the binding curves. (C) Left: a ribbon model of the CtpB V118Y mutant structure, which was crystallized in the same resting conformation as CtpBi. Omit electron densities (1.95 Å resolution, contoured at 1.2 σ) of the catalytic triad and of the introduced Tyr118 are shown together with the respective stick models. Interatomic distances among the Ser309-Lys334-Gln338 active site residues are given and reveal that the OH group of Ser309 is located too far away (4.6 Å) to setup a functional catalytic triad. Accordingly, the V118Y crystal structure supports the model that peptide binding to the PDZ domain is critical to remodel CtpB into its active form, whereas the composition of the proteolytic site does not define the functional states. Middle: a zoomed-in image of Tyr118 that occupies a large part of the peptide-binding cleft of the PDZ domain protruding toward the indicated carboxylate-binding loop. Corresponding omit electron densities are shown. (D) Comparison of the thermal motion factors of the PDZ domain of wild-type CtpB and R168A mutant. Both proteins crystallized in the same crystal form thus experiencing a similar crystal contact, indicated by the gray sphere.

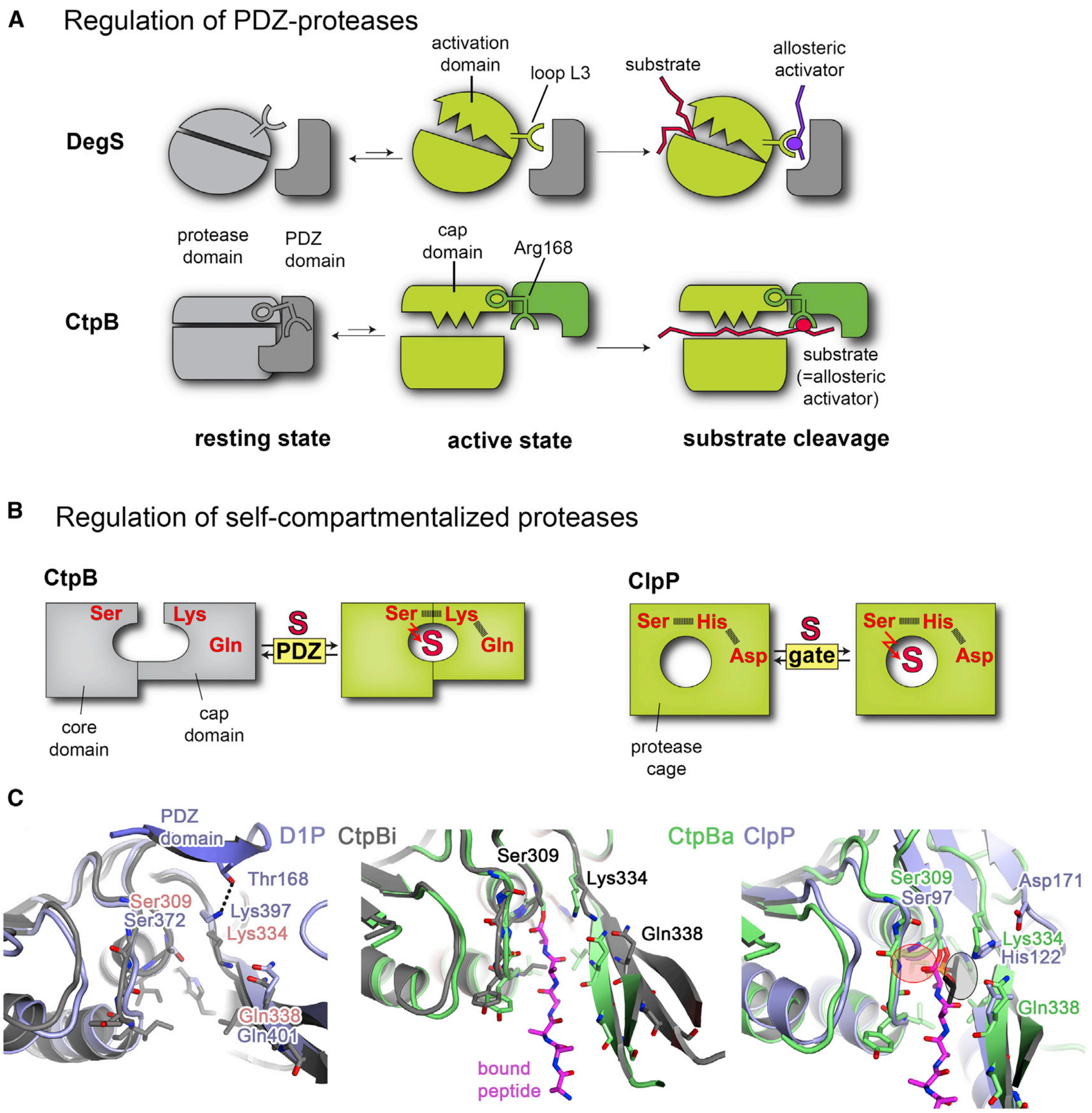

Figure S6. Mechanistic Implications from the Structural Studies of CtpB, Related to Figure 7

(A) Top: illustration of the similar concepts of allosteric regulation of the PDZ-proteases DegS and CtpB, despite belonging to distinct protease families. Both proteases are in equilibrium between resting and active conformations, and become stabilized in the active state once their PDZ domains bind an effector peptide. For this purpose, both proteases utilize a similar toolbox including a composite active site architecture that is composed of a protease core and an attached flexible domain (DegS: activation domain; CtpB: protease cap), and a signal sensing component (DegS: loop L3; CtpB: Arg168) that can read the peptide-binding state of the PDZ domain and stabilize the remodeled, functional proteolytic site in the presence of the allosteric activator. An important difference relates to the origin of the activator. In case of CtpB, substrate and activator are identical thus allowing the tight coupling of substrate binding and substrate cleavage. (B) Schematic presentation of the active site architecture of CtpB and ClpP illustrating their self-compartmentalizing character. Owing to the distinct origin of active site residues, CtpB is subject to allosteric regulation, whereas ClpP is remotely regulated by axial pores controlling substrate access to the protease cage. (C) Superposition of the active sites from D1P (blue), CtpBi (gray), CtpBa2 (green) and ClpP (blue) highlighting differences in the active site structure. Structural alignment of the protease cores yielded a root-mean-square deviation of 1.97 Å for CtpBa1-D1P, 1.67 Å for CtpBi-D1P, 1.94 Å for CtpBa1-ClpP and 2.36 Å for CtpBi-ClpP. According to these numbers, ClpP aligns better to the active conformation of CtpB. The peptide bound to CtpBa2 is shown as a stick model (pink) and the DFP bound to ClpP in black. The similarly arranged oxyanion hole (red circle) and S1 specificity pocket (black circle) are indicated together with residues of the respective catalytic triads. In contrast to ClpP, D1P aligns better to CtpBi, suggesting that D1P has been crystallized in an inactive conformation. While D1P and related proteases are reported to have a Ser/Lys catalytic diad (Keiler and Sauer, 1995; Liao et al., 2000), the similarity to CtpB suggests that the C-terminal processing proteases rather employ a Ser-Lys-Gln catalytic triad. Consistent with this, residue Gln338 is conserved among these proteases. Structural comparison reveals another interesting feature that may be relevant for regulating D1P. In the resting state of D1P, the catalytic Lys397 is captured by Thr168, a residue located next to the peptide-binding site of the PDZ domain. Therefore, peptide binding to the PDZ domain could break the interaction between Thr168 and Lys397 freeing Lys397 to setup a functional catalytic triad. Similarly to CtpB, such a scenario would link PDZ peptide binding with protease activation, however, rely on a different molecular mechanism.
